# Ninjurin 1 dodecamer peptide containing the N-terminal adhesion motif (N-NAM) exerts proangiogenic effects in HUVECs and in the postischemic brain

**DOI:** 10.1038/s41598-020-73340-5

**Published:** 2020-10-07

**Authors:** Seung-Woo Kim, Hye-Kyung Lee, Song-I. Seol, Dashdulam Davaanyam, Hahnbie Lee, Ja-Kyeong Lee

**Affiliations:** 1 Department of Anatomy, Medical Research Center, Inha University School of Medicine, Inharo 100, Inchon, 22202 Republic of Korea; 2 Medical Research Center, Inha University School of Medicine, Inchon, Republic of Korea; 3 Department of Biomedical Sciences, Inha University School of Medicine, Inchon, Republic of Korea

**Keywords:** Neuroscience, Diseases

## Abstract

Nerve injury-induced protein 1 (Ninjurin 1, Ninj1) is a cell adhesion molecule responsible for cell-to-cell interactions between immune cells and endothelial cells. In our previous paper, we have shown that Ninj1 plays an important role in the infiltration of neutrophils in the postischemic brain and that the dodecamer peptide harboring the Ninj1 N-terminal adhesion motif (N-NAM, Pro^26^-Asn^37^) inhibits infiltration of neutrophils in the postischemic brain and confers robust neuroprotective and anti-inflammatory effects. In the present study, we examinedt the pro-angiogenic effect of N-NAM using human umbilical vein endothelial cells (HUVECs) and rat MCAO (middle cerebral artery occlusion) model of stroke. We found that N-NAM promotes proliferation, migration, and tube formation of HUVECs and demonstrate that the suppression of endogenous Ninj1 is responsible for the N-NAM-mediated pro-angiogenic effects. Importantly, a pull-down assay revealed a direct binding between exogenously delivered N-NAM and endogenous Ninj1 and it is N-terminal adhesion motif dependent. In addition, N-NAM activated the Ang1-Tie2 and AKT signaling pathways in HUVECs, and blocking those signaling pathways with specific inhibitors suppressed N-NAM-induced tube formation, indicating critical roles of those signaling pathways in N-NAM-induced angiogenesis. Moreover, in a rat MCAO model, intranasal administration of N-NAM beginning 4 days post-MCAO (1.5 µg daily for 3 days) augmented angiogenesis in the penumbra of the ipsilateral hemisphere of the brain and significantly enhanced total vessel lengths, vessel densities, and pro-angiogenic marker expression. These results demonstrate that the 12-amino acid Ninj1 peptide, which contains the N-terminal adhesion motif of Ninj1, confers pro-angiogenic effects and suggest that those effects might contribute to its neuroprotective effects in the postischemic brain.

## Introduction

Angiogenesis in the postischemic brain is a complex process that results in the development of new vessels from pre-existing vasculature. The proangiogenic process progresses from hours to weeks after an ischemic injury, and growth factors such as vascular endothelial growth factor (VEGF) and Brain derived neurotrophic factor are known to play important roles in the survival of the component cells that compose neurovascular units in the penumbra of the postischemic brain^1,2^. Numerous studies have indicated that active angiogenesis can improve functional outcomes after ischemic stroke and accelerate brain recovery^3–5^ by removing damaged tissue^6^ and facilitating neural stem-cell migration^7,8^. On the other hand, immature vessels might exacerbate vascular and neuronal damage after ischemic stroke by bleeding into the brain^9^.

Ninjurin 1, nerve injury-induced protein 1 (Ninj1), is an adhesion protein that possesses two transmembrane domains, and an adhesion motif is located in its extracellular N-terminal region and known to be involved in homophilic interaction^10^. Ninj1 is widely expressed in various cell types. During development, it is expressed in perivascular macrophages near hyaloid vessels and involved in early ocular development^11^. In osteoclasts, Ninj1 participates in the regulation of bone homeostasis^12^. Ninj1 induction has also been reported under various pathological conditions, such as in hepatocellular carcinoma^13^ and acute lymphoblastic B-cell leukemia^14^, after cavernous nerve injury^15^, and in endometriosis^16^. In the peripheral nervous system, Ninj1 is up-regulated in neurons and Schwann cells after sciatic nerve injury and promotes nerve regeneration^10^. Ninj1 also participates in central nervous system (CNS) inflammatory processes, as shown in a mouse model of experimental autoimmune encephalomyelitis (EAE)^17^ and in T cell migration into the CNS, as shown in the same animal model^18^.

Functionally blocking Ninj1 has beneficial effects in various animal models of diseases. Ninj1 knockdown using siRNA or a neutralizing antibody restored erectile function by promoting the regeneration of penile endothelial and neuronal cells in a mouse model of diabetic erectile dysfunction^15^. Since Araki et al.^10^ reported that a peptide containing an adhesion motif, Pro^26^-Asn^37^, played a critical role in homophilic adhesion in an aggregation assay, several authors have reported that this region is important for the functional blocking of Ninj1. In particular, treatment with a peptide harboring this motif markedly reduced myeloid cell migration in an EAE animal model^17^ and activated T cells to enter the CNS^18^. In addition, an Ninj1-blocking peptide exerted anti-inflammatory effect in septic animal model^19^ and anti-apoptotic and anti-inflammatory effects on endothelial cells in an animal model of diabetes mellitus^20^. That blocking peptide also regulated cell proliferation and migration in a genetic status of p53-dependent manner, which is wild type or mutant^21^. Recently, we reported that Ninj1 was responsible for neutrophil infiltration and aggravated of inflammation in the postischemic brain and that functionally blocking Ninj1 using the N-terminal blocking peptide (Ninjurin 1 N-terminal adhesion motif (N-NAM, Pro^26^-Asn^37^)) suppressed inflammation and neuronal damage in the middle cerebral artery occlusion (MCAO) animal model of stroke^22^.

In the present study, we investigated the proangiogenic potential of N-NAM in HUVECs (human umbilical vein endothelial cells) and a rat model of ischemic stroke (MCAO). We found that N-NAM confers robust proangiogenic effects in HUVECs in an endogenous Ninj1-dependent manner. N-NAM binds directly with endogenous Ninj1, and the Ang1-Tie2 and AKT1 signaling pathways are involved in its proangiogenic effects. Furthermore, intranasal administration of N-NAM induced angiogenesis in the postischemic brain and significantly enhanced total vessel lengths, vessel densities, and proangiogenic marker expression.

## Results

### N-NAM induced the proliferation of HUVECs

**Figure 1:**
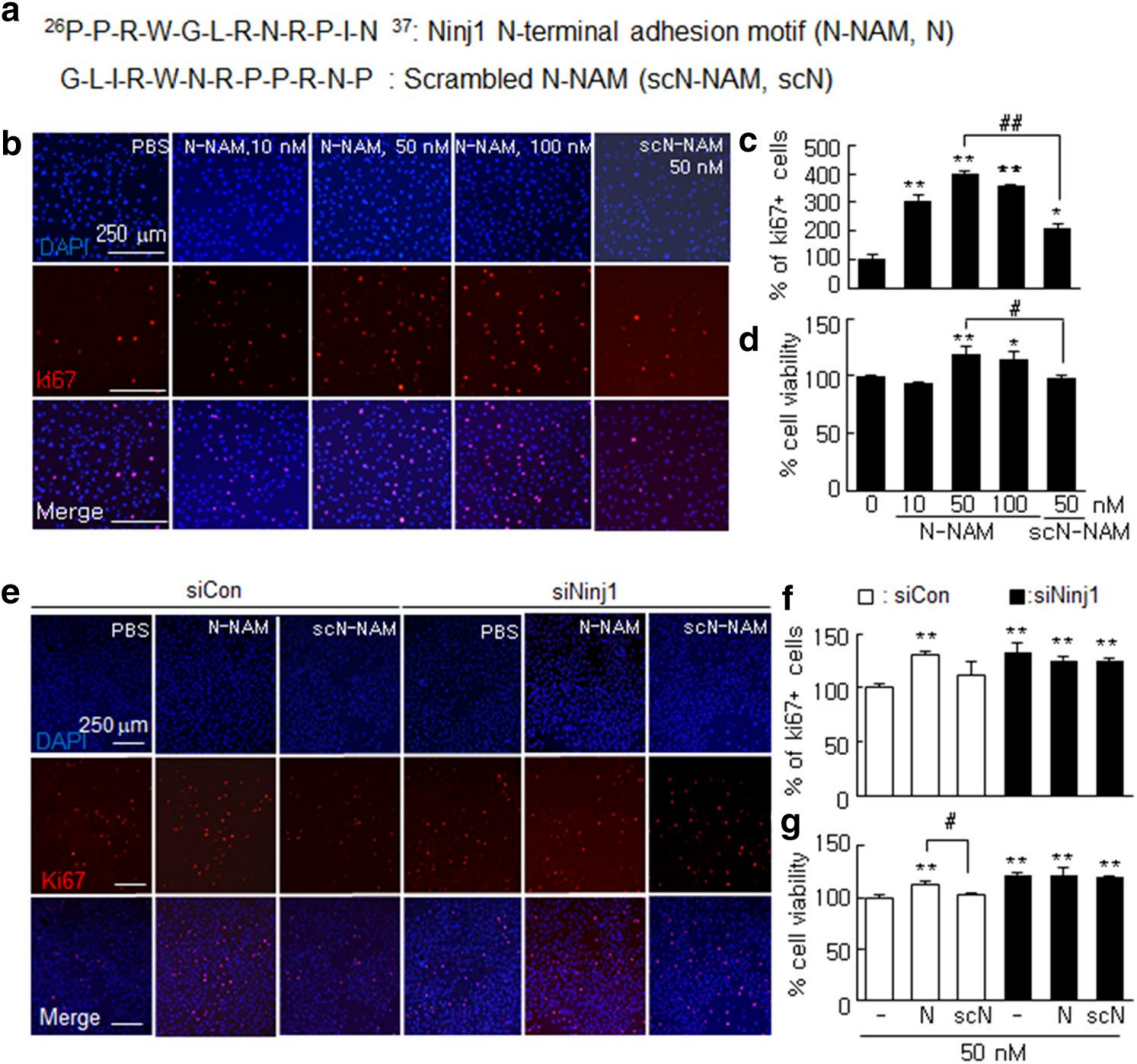
Induction of cell proliferation by N-NAM in human umbilical vein endothelial cell (HUVEC) cultures. (**a**) Sequences of N-NAM and scrambled N-NAM (scN-NAM). (**b**,**c**) HUVECs were treated with N-NAM (10, 50, 100 nM) or scN-NAM (50 nM) for 24 h, and cell proliferation was measured after immunofluorescent staining with anti-Ki67 antibody. Ki67-positive cells among DAPI (4′,6-diamidino-2-phenylindole)-positive cells were counted in two high-power fields in each of three plates. (**e**,**f**) Ninj1 siRNA (siNinj1) and non-specific siRNA (siCon) were transfected into separate HUVECs, and then the cells were treated with 50 nM of N-NAM or scN-NAM. The number of Ki67-positive cells was counted after immunostaining with anti-Ki67 antibody. Representative images are presented (**b**,**e**), and the results are presented as the means ± SEMs (n = 6). (**d**,**f**) MTT assays were performed under the same conditions used in b and e. The results are also presented as the means ± SEMs (n = 6). Scale bars, 250 µm. **p < 0.01, *p < 0.05 versus PBS-treated controls, ^##^*p* < 0.01, ^#^*p* < 0.05 between indicated groups.

To determine whether N-NAM (Fig. 1a) would induce endothelial cell proliferation, we treated HUVECs with 10, 50, or 100 nM of N-NAM or with 50 nM of scN-NAM (Fig. 1a) for 24 h, and then we counted the number of Ki67-positive cells. The numbers of Ki67-positive cells were increased by 207.1 ± 15.8% (n = 6) in the 10 nM N-NAM-treated cells compared with the PBS-treated controls, and the peak increase was detected with 50 nM N-NAM (300.0 ± 9.0%, n = 6) (Fig. 1b,c). To determine whether endogenous Ninj1 participated in the N-NAM-mediated induction of HUVEC proliferation, proliferation levels were examined after treating the cells with Ninj1 siRNA. The Ninj1 level was reduced to 22.3 ± 2.5% (n = 3) in cells transfected with Ninj1 siRNA, but it was unaffected in siCon-transfected cells (Supplementary Figure 1). Interestingly, HUVEC proliferation increased to 131.3 ± 7.4% (n = 6) in siNinj1-transfected cells and N-NAM treatment had no significant effect on that increase (125.4 ± 4.0%, n = 6) (Fig. 1e,f). In contrast, in the siCon-treated HUVECs, cell proliferation was unchanged but it increased significantly when the cells were co-treated with N-NAM (130.4 ± 3.9%, n = 6) (Fig. 1e,f). N-NAM-mediated HUVEC proliferation was confirmed by the MTT assay, which showed that cell survival was greater for N-NAM-treated cells than for scN-NAM-treated cells or PBS-treated control cells and the level increased significantly in the absence of endogenous Ninj1 (Fig. 1d,g). Together these results indicate that N-NAM induced HUVEC proliferation and that this induction required endogenous Ninj1.

### N-NAM induced HUVEC migration

**Figure 2:**
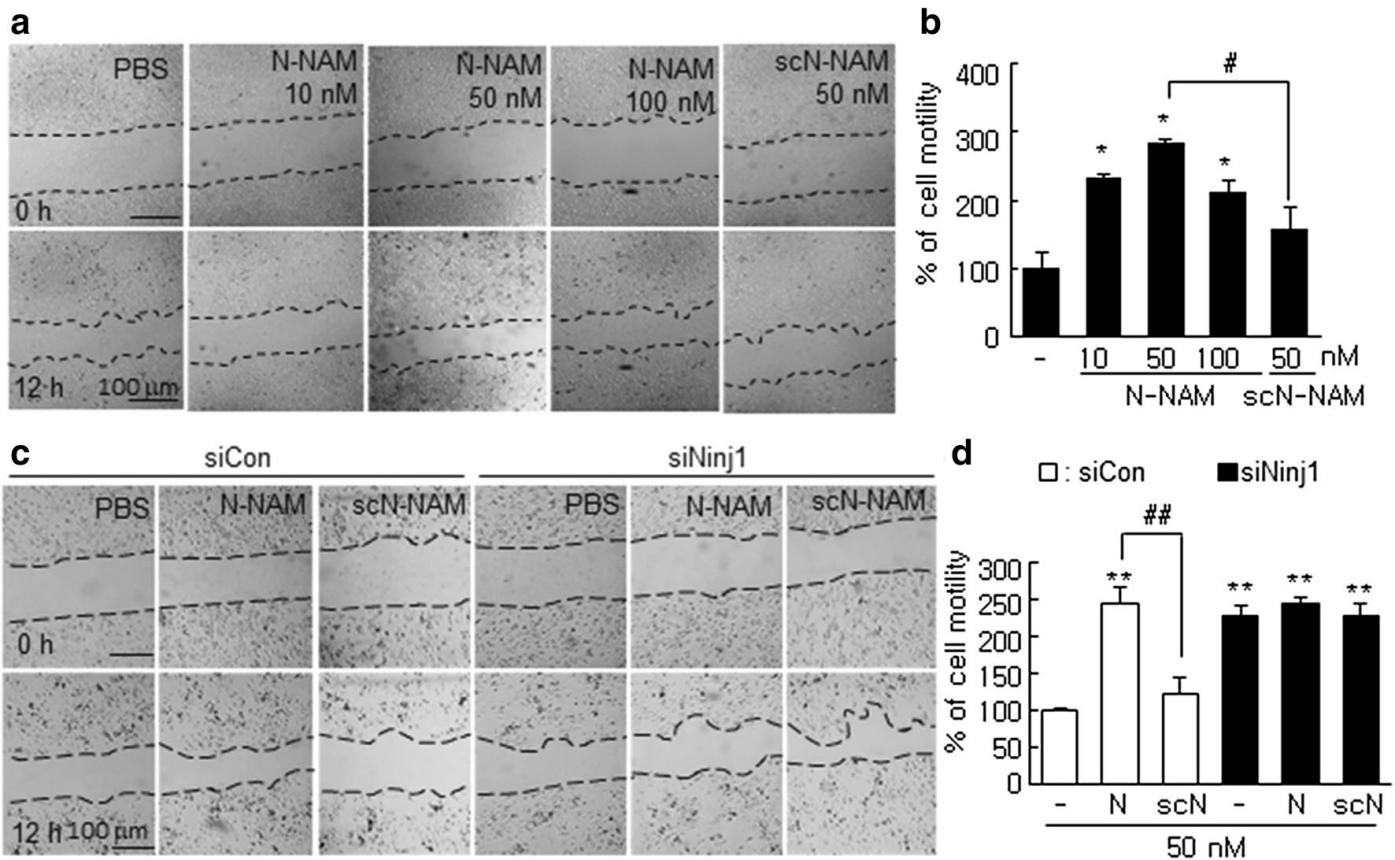
Induction of cell migration by N-NAM in HUVECs. (**a**,**b**) HUVECs were incubated with N-NAM (10, 50, or 100 nM) or scN-NAM (50 nM) for 12 h, and cell migration was evaluated using a wound healing assay and measuring wound widths at 0 and 12 h after scratching. (**c**,**d**) HUVECs were transfected with Ninj1 siRNA (siNinj1) or non-specific siRNA (siCon) and then treated with 50 nM of N-NAM or scN-NAM. Cell migrations was evaluated using a wound healing assay and measuring wound widths at 0 and 12 h. Representative images are shown (**a**,**c**), and the results are presented as the means ± SEMs (n = 5) (**b**,**d**). Scale bars, 100 μm. **p < 0.01, *p < 0.05 versus PBS-treated controls, ^#^*p* < 0.05, ^##^*p* < 0.01 between indicated groups.

To determine whether N-NAM would induce HUVEC migration, we used a wound healing assay after treating cells with N-NAM (10, 50, or 100 nM) or scN-NAM (50 nM) for 12 h. Cell motility, determined by measuring wound widths, increased with 10 nM N-NAM, peaking after treatment with 50 nM N-NAM at 341.0 ± 12.0% (n = 5) of the PBS-treated control (Fig. 2a,b). However, cell migration was not increased in the scN-NAM-treated group (Fig. 2a,b). Importantly, cell migration increased significantly to 228.0 ± 14.0% (n = 3), following siRNA-mediated Ninj1 knockdown (KD), with co-treatment with N-NAM failed to induce an additional effect (Fig. 2c,d). As no change in cell migration was detected after treating HUVECs with siCon, but co-treatment with N-NAM and siCon significantly increased cell migration to 247.0 ± 4.0% (n = 5) compared with the control (Fig. 2c,d). These results indicate that N-NAM induced HUVEC migration in an endogenous Ninj1-dependent manner.

### N-NAM induced HUVEC tube formation

**Figure 3:**
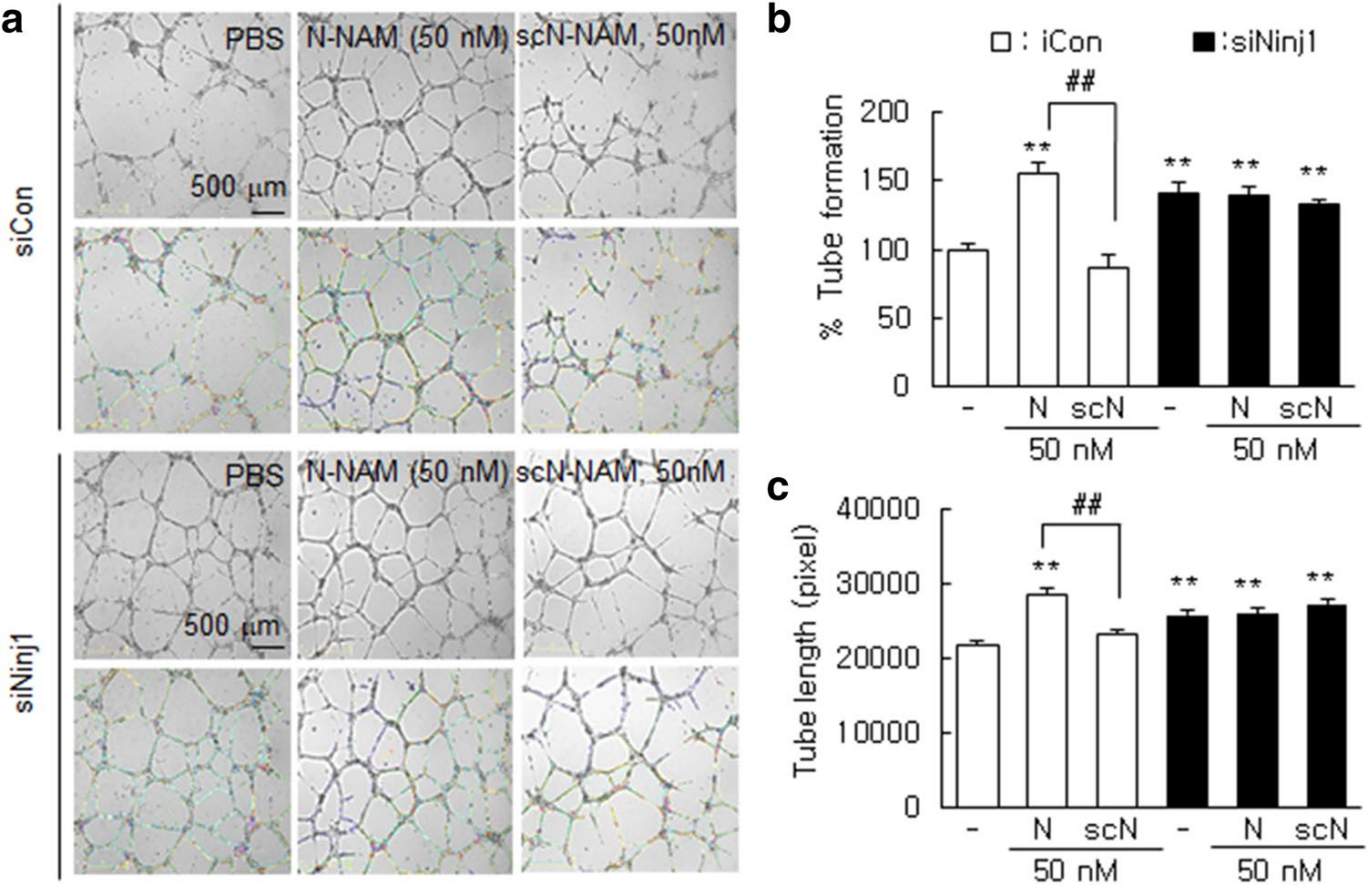
Induction of tube formation by N-NAM in HUVECs. HUVECs were transfected with Ninj1 or non-specific siRNA, and tube formation was accessed after incubating the cells with N-NAM (50 nM) or scN-NAM (50 nM) for 12 h. (**a**) Images obtained using an ImageJ analyzer (https://imagej.nih.gov/ij/download.html) are presented (green, branches; yellow, master segments; blue, tubes; red, master junctions) and (**b**) the number of tubes and (**c**) total tube lengths were measured. Results are presented as the means ± SEMs (n = 10). Scale bars, 500 μm. **p < 0.01 versus PBS-treated controls, ^##^*p* < 0.01 between indicated groups.

To confirm the proangiogenic effects of N-NAM, we examined tube formation by HUVECs. The induction of tube-like structures harboring branches, segments, and nodes was observed after culturing HUVECs on Matrigel for 12 h. The induction of tube formation was augmented dose-dependently by N-NAM, with 50 µMN-NAM increasing tube formation to 165.8 ± 5.9% (n = 10) versus the PBS-treated control cells, whereas scN-NAM had no such effect (Supplementary Figure 2). Similarly, the mean total tube length after 12 h of treatment was also increased significantly in the presence of N-NAM (Supplementary Figure 2). As expected, N-NAM significantly induced tube formation in siCon-transfected HUVECs (Fig. 3a,b). Tube formation was also enhanced to 166.3 ± 8.4% (n = 10) in Ninj1-KD HUVECs, but N-NAM-mediated enhancement of tube formation was not detected in the absence of endogenous Ninj1 (Fig. 3a,b). Similar results were obtained when total tube lengths were measured in Ninj1-KD HUVECs (Fig. 3a,c), indicating that N-NAM induced tube formation in HUVECs in an endogenous Ninj1-dependent manner.

### Proangiogenic effects of N-NAM in a reconstituted tissue model of angiogenesis

**Figure 4:**
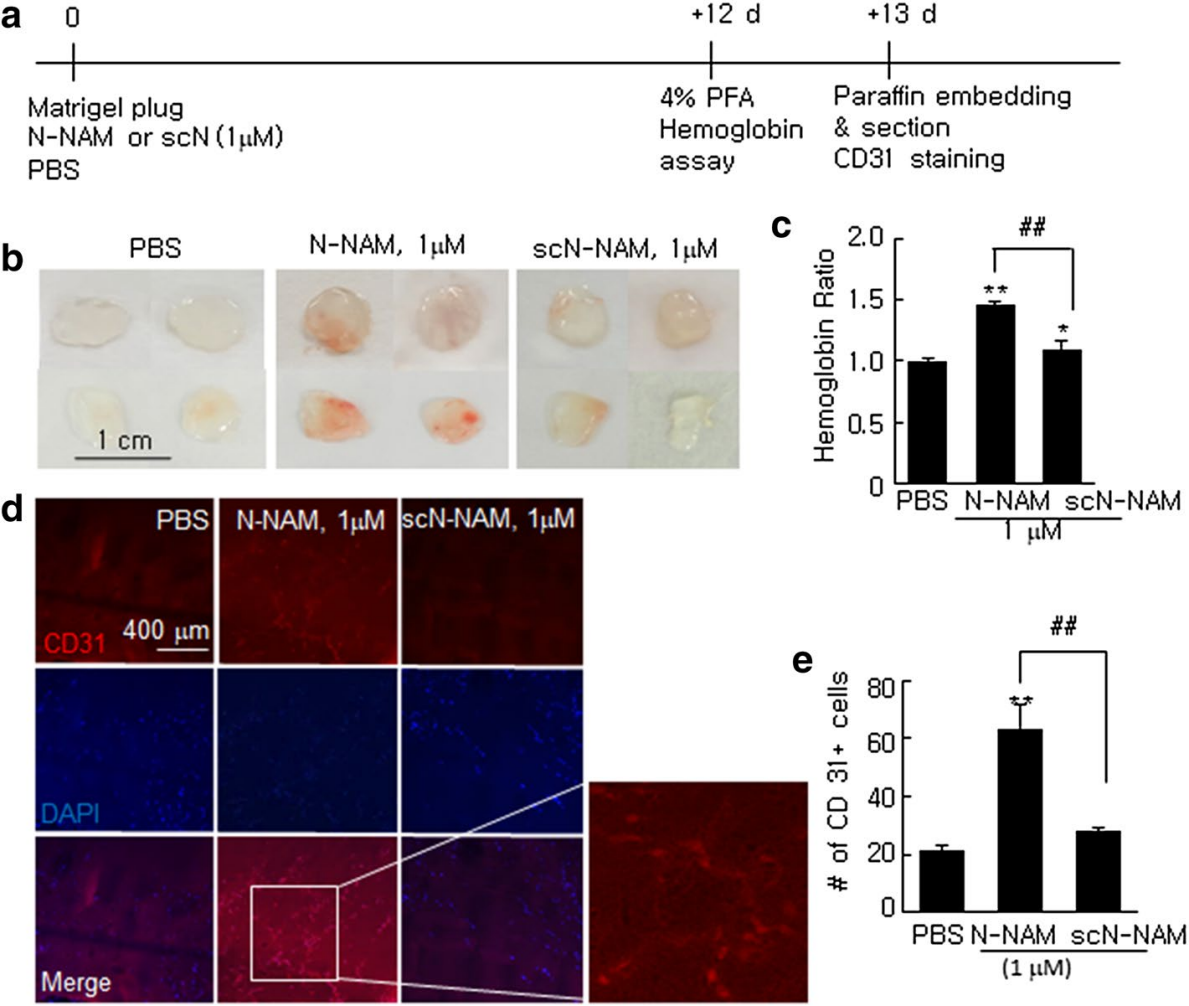
Proangiogenic effects of N-NAM in a reconstituted tissue model of angiogenesis. (**a**) Matrigel was mixed with 1 µM of N-NAM, scN-NAM, or PBS and injected subcutaneously into the mid-ventral region of BALB/c mice. Plugs were harvested 12 days later and subjected to a hemoglobin assay and immunofluorescent staining. (**b**) Representative images of Matrigel after harvesting. (**c**) Hemoglobin content was measured and normalized versus the Matrigel weights in the plugs. Results are presented as the means ± SEMs (n = 3). (**d**,**e**) Plugs were embedded in paraffin, and sections were prepared and stained with anti-CD31 antibody. The number of CD31-positive cells was counted and the results are presented as the means ± SEMs (n = 3). **p < 0.01, *p < 0.05 versus PBS-treated controls, ^##^*p* < 0.01 between indicated groups.

Next, we examined N-NAM-mediated angiogenesis using a mouse Matrigel Plug assay. Matrigel plugs containing N-NAM (1 µM) or scN-NAM (1 µM) were embedded under the skin in the mid-ventral region of BALB/c mice and harvested 12 days later (Fig. 4a). Massive neovascularization was detected in the N-NAM-containing plugs but not in the scN-NAM-containing plugs (Fig. 4b). When we measured the hemoglobin content in the plugs, we found significantly more hemoglobin in the N-NAM-containing plugs (148.2 ± 4.8% (n = 3) versus PBS-treated controls) and a moderate level of induction in the sc-N-NAM-containing plugs (Fig. 4c). When we counted the number of endothelial cells were counted in paraffin sections of the Matrigel plugs after staining them with anti-CD31 antibody (an endothelial marker), N-NAM-containing plugs had a significantly higher number of CD31^+^ cells than the control, but the scN-NAM-containing plugs did not (Fig. 4d,e). These results further confirmed the pro-angiogenic effect of N-NAM.

### Interaction between N-NAM and endogenous Ninj1 and induction of proangiogenic markers in HUVECs in a Ninj1 N-terminal-dependent manner

**Figure 5:**
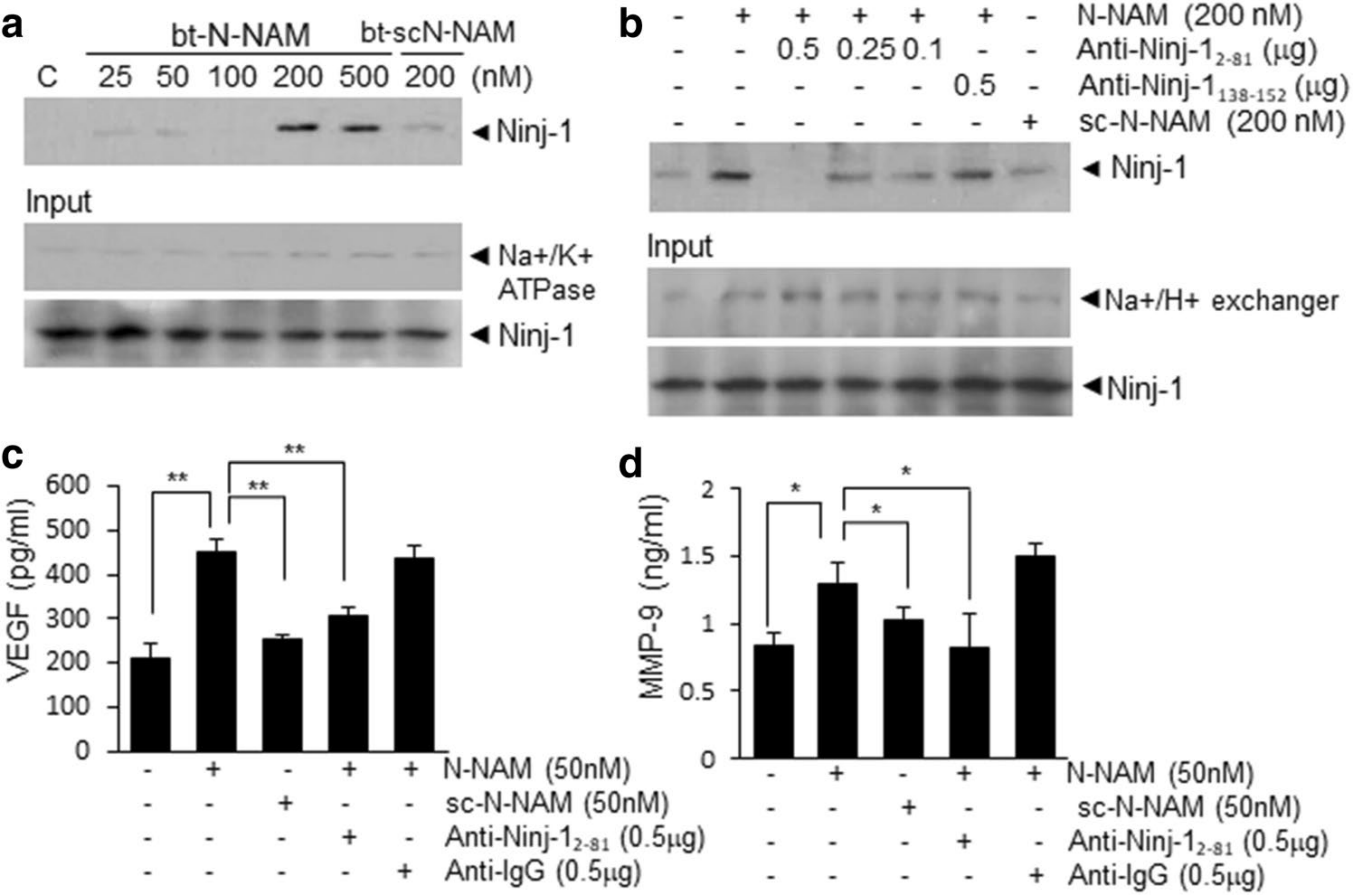
Interaction between N-NAM and endogenous Ninj1 and induction of proangiogenic markers in HUVECs. Direct binding between endogenous Ninj1 and biotinylated-N-NAM (bt-N-NAM) or biotinylated-scN-NAM (bt-scN-NAM) was examined using a pull-down assay. (**a**) HUVECs were treated with bt-N-NAM (25, 50, 100, 200, or 500 nM) or bt-scN-NAM (200 nM) for 3 h. Complexes were pulled down with streptavidin beads, and the level of Ninj1 in each binding complex was measured by immunoblot using anti-Ninj1 antibody. (**b**) HUVECs were preincubated with anti-Ninj1_2–81_ (0.1, 0.25, or 0.5 ng/μl) or anti-Ninj1_138–152_ antibody (0.5 ng/μl) for 15 min and then treated with bt-N-NAM (200 nM) or bt-scN-NAM (200 nM) for 3 h and a pull-down assay was carried out as described in (**a**). A Na^+^/K^+^ ATPase , Na^+^/H^+^ exchanger, and Ninj1 were used as input controls. (**c**,**d**) VEGF (vascular endothelial growth factor) and MMP9 (matrix metallopeptidase 9) levels in culture media from HUVECs were assessed by ELISA after treating the cells with N-NAM (50 nM) or scN-NAM (50 nM) in the presence or absence of anti-Ninj1_2–81_ (1 µg/ml) antibody or IgG (1 µg/ml). Results are presented as the means ± SEMs (n = 3). **p < 0.01, *p < 0.05 versus the N-NAM-treated cells.

To investigate whether N-NAM binds endogenous Ninj1, a pull-down assay was performed using biotinylated-N-NAM (bt-N-NAM). The results demonstrate the interaction between bt-N-NAM and endogenous Ninj1 in bt-N-NAM-treated HUVECs; however, that interaction was not detected when bt-scN-NAM was used (Fig. 5a). Interestingly, the interaction was also undetectable when the HUVECs were preincubated with N-terminal-specific Ninj1 antibody (anti-Ninj1_2–81_ antibody). Furthermore, the inhibitory effects were dose-dependent on the anti-Ninj1_2–81_ antibody (Fig. 5b). Importantly, preincubation with Ninj1 C-terminal-specific antibody (anti-Ninj1_138–152_ antibody) had no effect on the binding between bt-scN-NAM and endogenous Ninj1 (Fig. 5b). In addition, in the media of N-NAM-treated HUVECs, the VEGF and MMP9 protein levels (proangiogenic markers) were significantly higher than in PBS-treated controls, however, those enhancements were not observed in scN-NAM-treated cells (Fig. 5c,d). Moreover, the induction of VEGF and MMP9 did not occur when cells were preincubated with anti-Ninj1_2–81_ antibody and then treated with N-NAM (Fig. 5c,d). These results indicate that the N-terminal region of Ninj1 is critical for the binding between exogenous N-NAM and endogenous Ninj1 and the proangiogenic effects of N-NAM.

### The Ang1-Tie2 and AKT signaling pathways were involved in the proangiogenic effects of N-NAM in HUVECs

**Figure 6:**
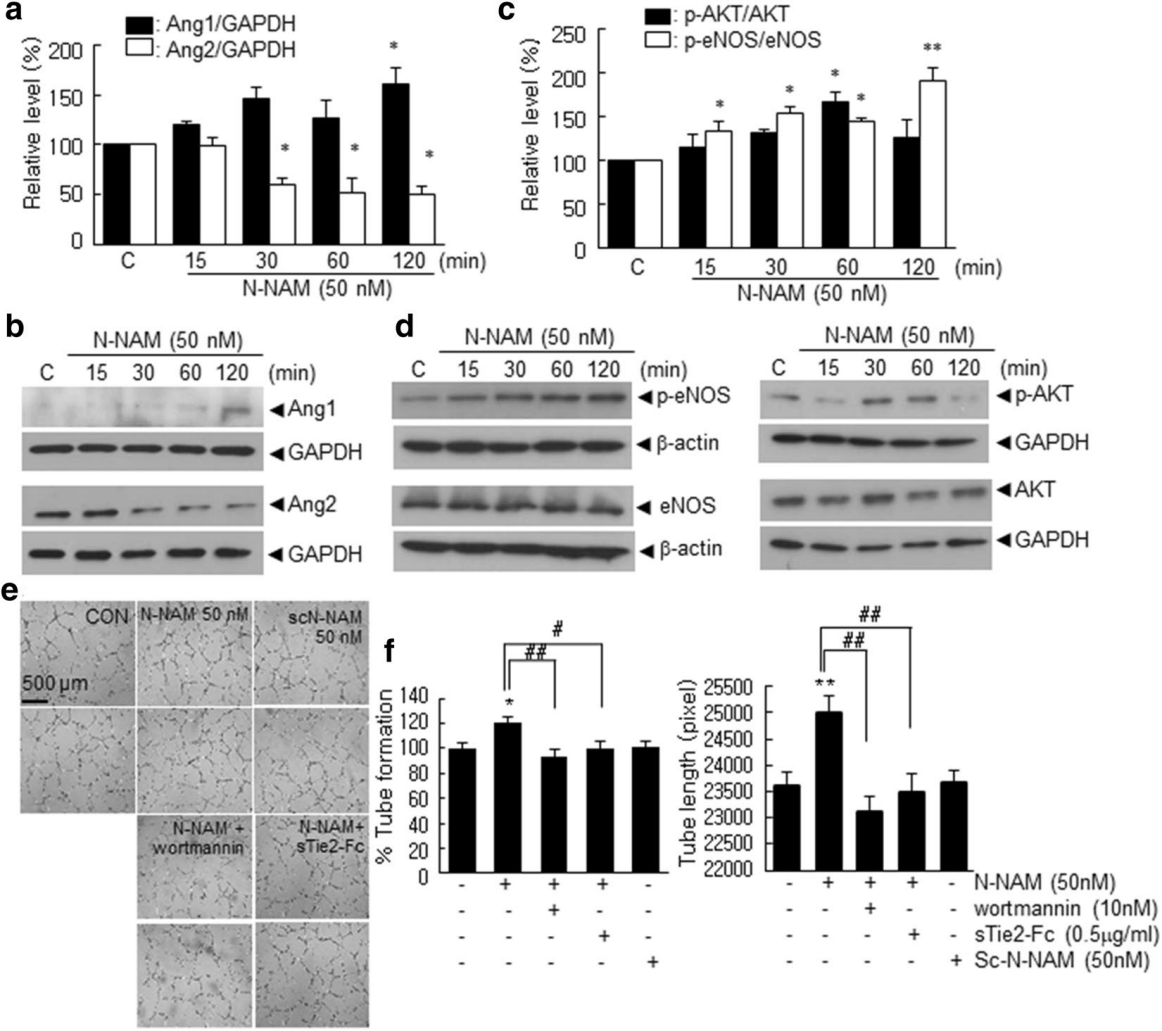
The Ang1-Tie2 and PI3K/AKT signaling pathways were involved in the N-NAM-mediated proangiogenic effect. (**a**–**d**) HUVECs were incubated with N-NAM (50 nM) for 15, 30, 60, or 120 min, and Ang1 or Ang2 levels (**a**,**b**) and total and phosphorylated AKT and eNOS levels (**c**,**d**) were assessed by immunoblotting. Representative images are presented (**b**,**d**) and results are presented as the means ± SEMs (n = 3 for Ang1, Ang2, and eNOS and n = 5 for AKT) (**a**,**c**). (**e**–**f**) HUVECs were treated with N-NAM (50 nM) or scN-NAM (50 nM) for 12 h with or without sTie2-Fc (0.5 µg/ml) or wortmannin (10 nM) pretreatment and tube formation was examined. Representative images are presented and the number of tubes and total tube lengths are presented as the means ± SEMs (n = 10). Scale bar in e, 500 µm, **p < 0.01, *p < 0.05 versus the PBS-treated controls, ^#^*p* < 0.05 versus 50 nM N-NAM-treated cells.

Expression of angiopoietin-1, angiopoietin-2, and tie receptors has been reported to play important role in angiogenesis in the ischemic brain^23^ and in particular, angiopoietin-1 inhibits endothelial cell apoptosis via the AKT/survivin pathway^24,25^. Western blot analyses showed that the Ang1 level increased significantly, and the Ang2 level decreased gradually and significantly in N-NAM (50 nM)-treated HUVECs (Fig. 6a,b). In addition, phospho-AKT (Ser473) and phospho-eNOS (Ser1177) levels were also significantly increased after N-NAM (50 nM) treatment (Fig. 6c,d). To further confirm the importance of the above mentioned signaling in the proangiogenic effect of N-NAM, tube formation was examined after pretreating HUVECs with sTie2-Fc, the soluble extracellular domain of the Tie2 receptor, or with wortmannin. N-NAM-mediated tube formation was suppressed in a dose-dependent manner (Supplementary Figure 3) and decreased to almost basal levels by 0.5 µg/ml sTie2-Fc and 10 nM wortmannin, a PI3K inhibitor (Fig. 6e,f). Taken together, these results indicate that the Ang1-Tie2 and AKT signaling pathways play important roles in N-NAM-mediated tube formation in HUVECs.

### Proangiogenic effects of N-NAM in the postischemic brain

**Figure 7:**
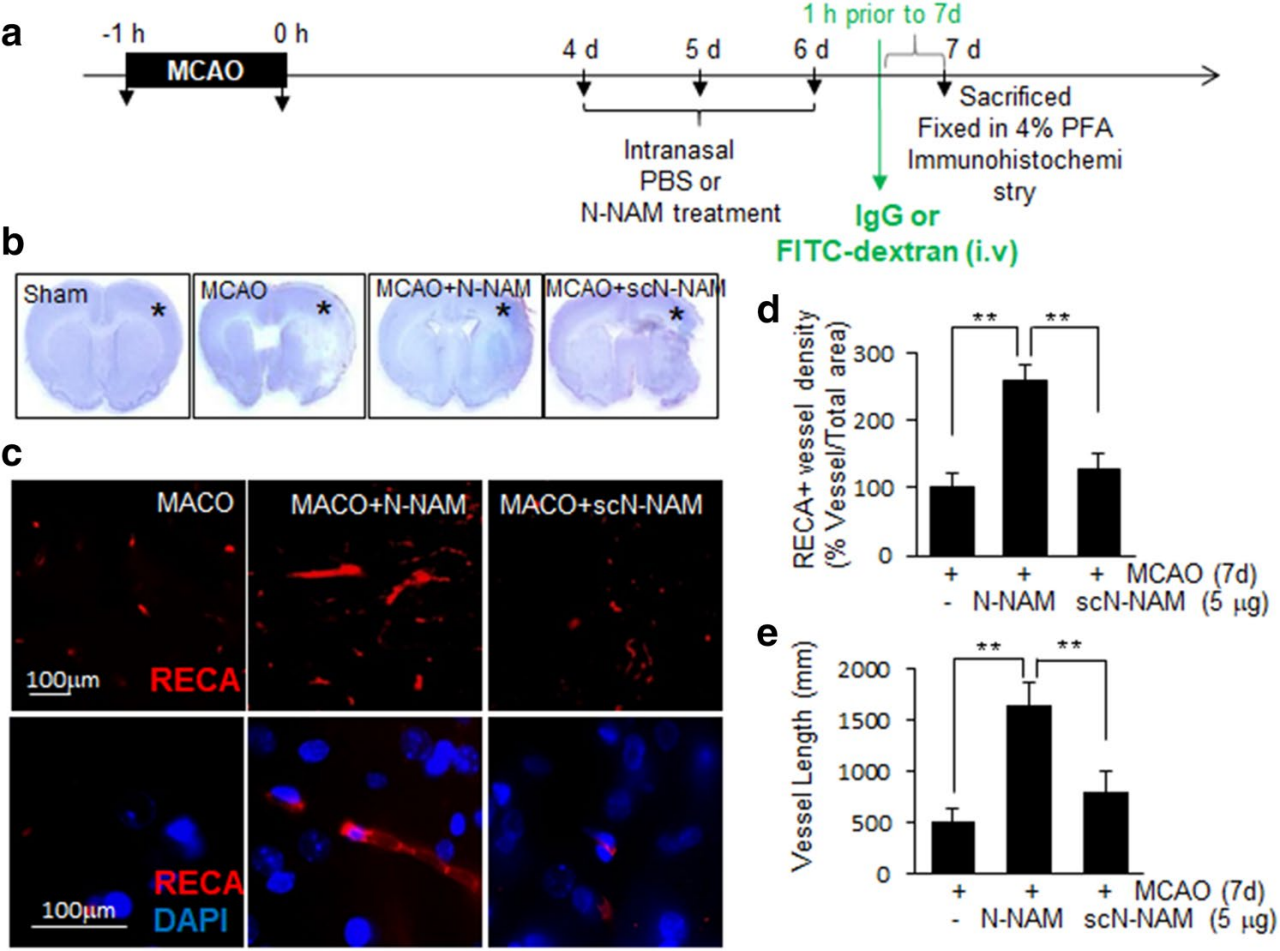
Proangiogenic effects of N-NAM in the postischemic brain. (**a**) N-NAM (5 µg) or scN-NAM (5 µg) was administered intranasally three times daily at 4, 5, and 6 days after 60 min of MCAO. (**b**,**c**) At 7 days post-MCAO, coronal brain sections were stained using cresyl violet (**b**) or double-fluorescence-stained with anti-rat endothelial cell antigen-1 (RECA-1) antibody and DAPI (**c**). Representative images are presented (**c**), and RECA-1-positive vessel densities are presented as the mean ± SEMs (n = 5) (**d**). Total vessel lengths were measured using AngioTool software 0.6a (https://angiotool.software.informer.com/0.6/), and the results are presented as the means ± SEMs (n = 5) (**e**). Scale bars, 100 μm, **p < 0.01 versus the 50 nM N-NAM-treated group.

Next, we investigated proangiogenic effects of N-NAM in the postischemic brain using a rat model of cerebral ischemia. In our previous report, we showed a robust neuroprotective effect of N-NAM when it was administered earlier than 9 h post-MCAO^26^. To show the direct proangiogenic effect of N-NAM not an indirect outcome of the neuroprotective effect, N-NAM (5 μg) was administered intranasally 4, 5, and 6 days post-MCAO, and vessel formation was examined 7 days post-MCAO (Fig. 7a). Cresyl violet staining conducted at 7 days post-MCAO showed no difference in the infarct volume in the MCAO + N-NAM compared to that in MCAO + scN-NAM (Fig. 7b). When blood vessel densities were measured in the cortical penumbra of the ipsilateral hemispheres (Fig. 7b, asterisk) using an anti-RECA-1 antibody (a marker of endothelial cells), RECA-1-positive vessels were detected in the MCAO + PBS controls 7 days after MCAO (Fig. 7c). Importantly, vessel densities was significantly enhanced in the MCAO + NAM animals (259.1 ± 24.4% (n = 5) versus MCAO + PBS controls) but not in the MCAO + scN-NAM animals (Fig. 7c,d). Similarly, total vessel length was also markedly greater in the MCAO + N-NAM group (324.7 ± 42.4% (n = 5) of the MCAO + PBS controls) but not in the MCAO + scN-NAM animals (Fig. 7c,e). Together, these results demonstrate a robust proangiogenic effect of N-NAM in the postischemic brain.

### Attenuated vessel leakage and upregulation of angiogenesis marker induction by N-NAM in postischemic brains

**Figure 8:**
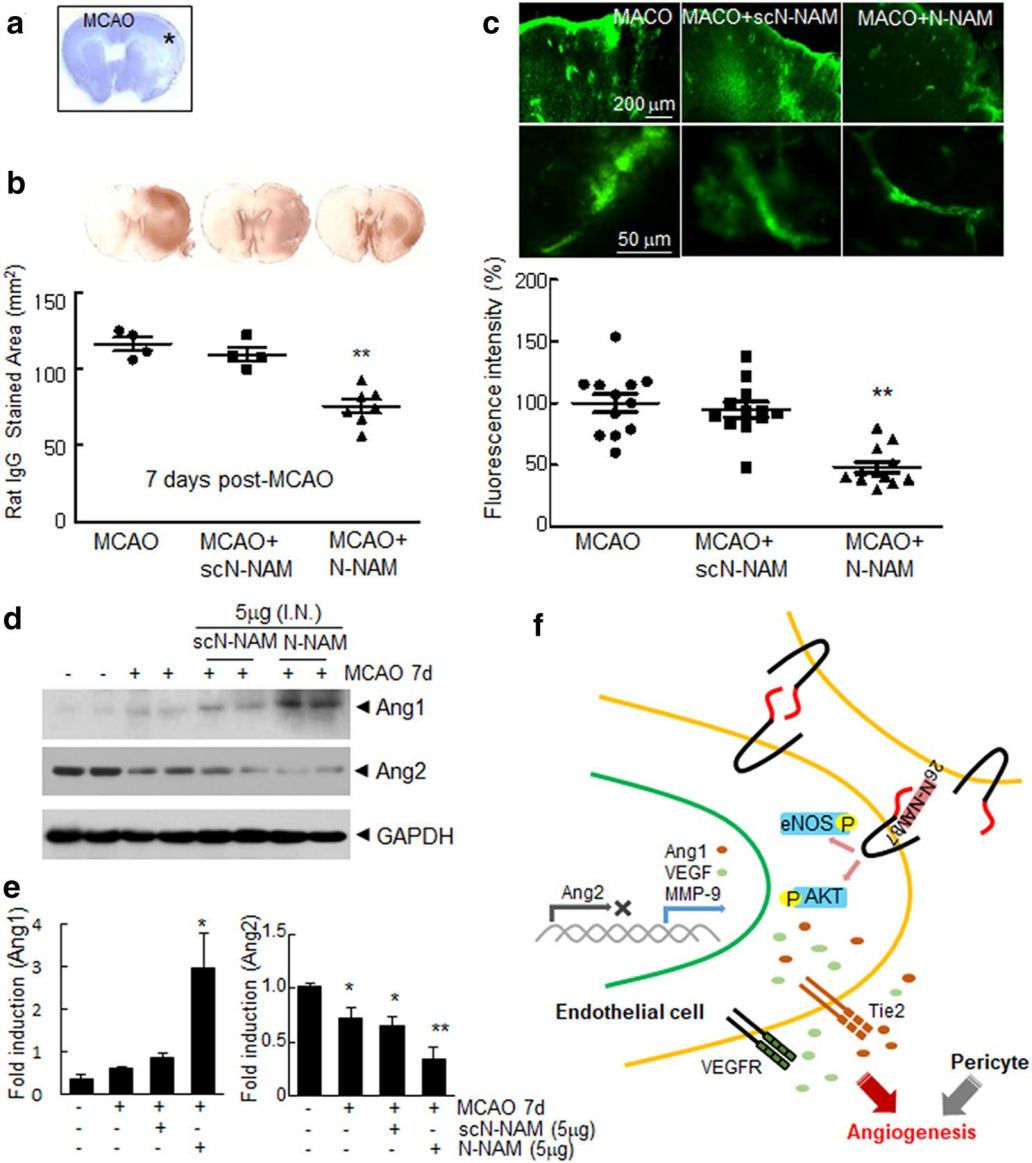
Functional blood vessel formation and proangiogenic marker induction by N-NAM in the postischemic brain. N-NAM (5 µg) or scN-NAM (5 µg) was administered intranasally three times daily at 4, 5 and 6 days after 60 min of MCAO, and IgG or fluorescein isothiocyanate (FITC)-dextran (60 mg/kg) was injected intravenously (i.v.) 1 h prior to sacrifice 7 days post-MCAO (Fig. 7a). Coronal brain sections were prepared and stained using biotynylated rat anti-IgG antibody for IgG staining (**b**) or FITC-dextran images acquired with confocal microscopy (**c**). IgG-positive area or FITC intensity were measured using Scion Image 4.0 (https://scion-image.software.informer.com/) or ImageJ (https://imagej.nih.gov/ij/download.html), respectively. Representative images are shown (upper panels in **b** and **c**), and the intraparenchymal leakage area are presented as the means ± SEMs (n = 7 for IgG staining and n = 12 (3 consecutive planes from 4 animals) for FITC-dextran image). (**d**,**e**) Tissue lysates were prepared from the asterisked regions in (**a**) 7 days post-MCAO and immunoblotted for Ang1 and Ang2. (**f**) Schematic diagram of a proposed mechanism showing how N-NAM induce angiogenesis in HUVECs. Ang1, angiopoietin-1; Ang2, angiopoietin-2; MMP-9, metalloprpoteinase 9; VEGF, vascular endothelial growth factor; eNOS, endothelial nitric oxide synthase. The results are representative of three independent experiments. Scale bars, 200 or 50 μm in C, **p < 0.01, *p < 0.05 versus theN-NAM + PBC control group.

To determine whether N-NAM induced functional blood vessel formation, we tested the permeability of the vessels using staining after administrating IgG or fluorescein isothiocyanate (FITC)-dextran. IgG or FITC-dextran was administered 1 h prior to sacrifice at 7 days post-MCAO (Fig. 7a). When we administered IgG and measured the intraparenchymal leakage area in the cortical penumbra of the ipsilateral hemisphere (asterisk in Fig. 8a), it was reduced in MCAO + N-NAM group to 75.4 ± 4.2% (n = 7) of the total area in MCAO + PBS control animals (Fig. 8b). In addition, after injecting FITC-dextran, the percent-leakage-area in the MCAO + N-NAM group was significantly decreased, 48.0 ± 8.5% (n = 12) of that in the MCAO + PBS controls (Fig. 8c), indicating that leakage from the new vessel was attenuated by N-NAM. In addition, the levels of Ang1 and Ang2 were significantly up- or down-regulated, respectively, in the cortical penumbra of the ipsilateral hemispheres in the MCAO + N-NAM group 7 days post-MCAO, but not in the MCAO + scN-NAM group (Fig. 8d,e). These results confirmed the proangiogenic potency of N-NAM in the postischemic brain.

## Discussion

In a previous study, we reported that Ninj1 was up-regulated during the acute phase after MCAO^22^ and that intranasally administered N-NAM had robust neuroprotective and anti-inflammatory effects in the same rat MCAO model^26^. In the present study, we have shown that N-NAM has proangiogenic effects on HUVECs and in the postischemic brain. HUVEC proliferation, migration, and tube formation were promoted by N-NAM, and those effects were accompanied by the up- or down-regulation of Ang1 and Ang2, respectively, and the induction of proangiogenic markers such as VEGF and MMP9 (Fig. 8f). Moreover, the intranasal administration of N-NAM as late as 4 days post-MCAO conferred a robust proangiogenic effect, as evidenced by increased vessel formation and the expression of proangiogenic markers. Although a few previous papers have reported the importance of the N-terminal Ninj1 peptide under various pathological conditions, this is the first report to show that it plays a critical role in angiogenesis. Furthermore, we demonstrated that this effect is due to inhibition of the suppressive effect that endogenous Ninj1 has on angiogenesis.

The proangiogenic effect found in our siRNA-mediated Ninj1 KD experiments indicates that endogenous Ninj1 has a negative effect on angiogenesis, which is in keeping with a previous report that angiogenesis was enhanced after functionally blocking Ninj1 using a neutralizing antibody or siRNA in diabetic mice^15^. In addition, increase in the function and survival of endothelial cells following treatment with a Ninj1 antibody was reported in a similar diabetic animal model^20^. In the present study, we found that endogenous Ninj1 KD enhanced angiogenesis and that in that situation, N-NAM exerted no angiogenic effect, which suggested that the proangiogenic effects of N-NAM resulted from the suppression of endogenous Ninj1. In the postischemic brain, penumbral endothelial cells begin to proliferate after 12–24 h and active angiogenesis is observed 3–4 days post-stroke^4,25^. It is interesting to note here that in our previous reports, we showed a dual surge of Ninj1 expression in the postischemic brain, one at ~ 12 h and another 4–6 days post-MCAO^22,26^. The neuroprotective effect of N-NAM administered immediately after MCAO, shown in our previous study^26^ was attributed to blocking Ninj1 from ~ 12 h, which resulted in the inhibition of neutrophil infiltration during the acute phase. On the other hand, proangiogenic effect of delayed N-NAM administration, beginning 4 days post-MCAO in the present study, might be derived from functional blocking of the 2nd surge of Ninj1 in the postischemic brain, which occurs from 4–6 days post-MCAO. Because that 2nd surge of Ninj1 decreased gradually but was maintained until 10 days post-MCAO in both the cortical and striatal penumbrae^22^, N-NAM might exert other effects, in addition to proangiogenic effects, related to the repair and reconstruction of the postischemic brain. Further studies are required on this topic.

In our previous reports, we showed that the neuroprotective and anti-inflammatory effects of N-NAM in the postischemic brain were due to the inhibition of interactions between neutrophils and endothelial cells^26^. We observed a direct interaction between N-NAM and endogenous Ninj1 in this study, so we suggested that functional blocking of the homophilic binding of Ninj1 via competitive binding underlies the proangiogenic effects of N-NAM. However, we cannot exclude the possibility that functional blocking of the heterophilic interactions between Ninj1 and another molecule (not yet identified) also play a role or that N-NAM could act as a novel ligand for another receptor. Furthermore, because the angiogenesis-related effects of Ninj1 have been reported in cells other than endothelial cells, for example in pericytes^27,28^ and macrophages^11^, it is possible that N-NAM also affects those cells. Although we failed to find any functional motif or proteins harboring sequences homologous to N-NAM in a BLAST search, additional studies are required to investigate the heterophilic interactions of Ninj1 by N-NAM and possible novel roles of N-NAM.

In this study, we have demonstrated that the Ang1-Tie2 and AKT signaling pathways are critically involved in the proangiogenic effects of N-NAM. Ninj1-KD-mediated Ang1-Tie2 activation and its proangiogenic effects in endothelial cells were previously reported in a diabetic mouse model^15^. Knockdown of the Ninj1 with siRNA increased Ang1 expression and decreased the endogenous Ang1 antagonist, Ang2, in cavernous endothelial cells in diabetic mouse which resulted in increased endothelial cell proliferation and decreased apoptosis^15^. Interestingly, similar modulation of Ang1-Tie2 signaling by Ninj1 has also been documented in pericytes^11^. During early ocular development, Ninj1-overexpressing macrophages decreased Ang1 and increased Ang2 in pericytes, resulting in hyoid vessel endothelial cell apoptosis^11^. Furthermore, in hind limb ischemia animal model, Ninj1 was induced in pericytes and modulated Ang1/Ang2 expression^27,28^. Pericytes play a critical role during the initial endothelial cell sprouting, guiding newly formed vessel^29^. Later, during the vascular maturation, pericytes are strongly associated with immature vessels and induces vascular stabilization and maturation^30,31^. Ninj1 in pericyte is involved in the vessel maturation through the association between pericytes and endothelial cells, leading to blood flow recovery from ischemia^28^. In the present study, we reported that N-NAM up- and down-regulates Ang1 and Ang2, respectively, in HUVECs and promotes vascular permeability in the postishcemic brain. Therefore, the contribution and modulation of Ang1-Tie2 signaling pathway in the presence of both endothelial cells and pericytes might provide more information and needs further study. However, although we observed direct binding between N-NAM and endogenous Ninj1, the link between such binding and the activation of AKT signaling was not established. Given that the cytoplasmic domain of Ninj1 is too small to directly activate downstream signaling molecules, it has been speculated that Ninj1 might be involved in the clustering of membrane proteins such as integrins and that those complexes might mediate signaling pathways in the cytoplasm^32,33^. Further studies are required to elucidate the downstream signaling of Ninj1 by which N-NAM regulates angiogenesis.

Although ischemic stroke is a major cause of death and disability, few therapeutic options are available, and only a small proportion of stroke patients receive acute reperfusion therapies. Nevertheless, as the survival rate has increased, long-term treatments have become necessary to address the sequelae of stroke. Therefore, researchers have begun to focus on recovery after stroke, including a search for drugs and treatments that promote functional recovery. Given the importance of angiogenesis during recovery, N-NAM might be a useful starting point for the development of therapeutics that target neuro-recovery from postischemic damage. Attenuation of delayed inflammation by N-NAM may also contribute to promote functional recovery following cerebral ischemia since numerous reports, including ours, have demonstrated the anti-inflammatory effects of this peptide^17–20,22^. It is important to note here that since in the process of reparative angiogenesis, the interplay between the immune cells and growing blood vessels is important, pro-angiogenic effect is associated with anti-inflammatory effect if the postischemic brain. Moreover, because it has been shown that Ninj1 is a multifunctional protein involved not only in vessel formation but also in nerve regeneration and cell–cell interactions under various pathological conditions^10,34–37^, N-NAM might affect repair process in the postischemic brain, such as neurite outgrowth or synaptogenesis.

## Conclusion

The present study showed the 12-amino acid Ninj1 peptide, which contains the N-terminal adhesion motif of Ninj1, confers pro-angiogenic effects and suggest that those effects might contribute to its neuroprotective effects in the postischemic brain. Thus inhibition of Ninj1 using N-NAM might be a promising approach to ameliorate delayed damage and enhance reparative responses. Additional studies are required to access the effect of N-NAM modulating the cross-talk among those multifunction and to clarify the N-NAM-induced signaling pathways in vivo wherein the interactions among endothelial, pericytes, and immune cells are allowed.

## Materials and methods

### Peptides

A peptide identical to the adhesion domain of Ninj1 (rat sequence: PPRWGLRNRPIN, human sequence: PARWGWRHGPIN) (PEPTRON, Daejeon, Korea) was used as a blocking peptide (N-NAM). A scrambled peptide (rat sequence: GLIRWNRPPRNP, human sequence: GLIRWNRPPRNP, scN-NAM) (PEPTRON, Daejeon, Korea) was used as the negative control in vivo and in vitro. Anti-Ninj1_2–81_ (R&D Systems, Minneapolis, MN)_,_ anti-Ninj1_138–152_ (MyBioSource, San Diego, CA), and control IgG (Santacruz, Santa Cruz Biotechnology, Santa Cruz, CA) were used blocking antibodies for functional assays.

### Surgical procedure used for MCAO

Male Sprague–Dawley rats were housed under diurnal lighting conditions and allowed food and tap water ad libitum. All animal studies were carried out in strict accordance with the Guide for the Care and Use of Laboratory Animals published by the National Institute of Health (2013) and the ARRIVE guidelines (https://www.nc3rs.org/ARRIVE). The animal protocol used was reviewed and approved by the Institutional Animal Care and Use Committee of INHA University (Approval Number INHA-160824-434). MCAO was carried out as described previously^26^. Briefly, 8-week-old male Sprague-Dawley rats (250–300 g) were anesthetized with 5% isoflurane in 30% oxygen/70% nitrous oxide and maintained using 0.5% isoflurane in the same gas mixture during surgery. Occlusion of the right middle carotid artery was induced for 1 h by advancing a nylon suture (4-0; AILEE, Busan, Korea) with a heat-induced bulb at its tip (~ 0.3 mm in diameter) along the internal carotid artery for 20–22 mm from its bifurcation with the external carotid artery. This procedure was followed by reperfusion for up to 7 days. A thermoregulated heating pad and a heating lamp were used to maintain a rectal temperature of 37 ± 0.5 °C during surgery. Animals were randomly allocated to the sham, MCAO + PBS (phosphate-buffered saline), MCAO + N-NAM, or MCAO + scN-NAM group. Animals in the sham group underwent an identical procedure, but the MCA was not occluded.

### Peptide treatment in vivo

Rats were anesthetized intramuscularly by injecting a mixture of ketamine (3.75 mg/100 g body weight) and xylazine hydrochloride (0.5 mg/100 g body weight). Peptides (5 μg) were dissolved in 10 μl of PBS (0.01 M) and injected intranasally 4, 5, and 6 days after MCAO. Brain tissues were collected 7 days after MCAO.

### HUVEC cultures

Human umbilical vein endothelial cells (HUVECs) were purchased from the American Type Culture Collection (ATCC; Manassas, VA). Cells were cultured on diverse culture dishes coated with 0.1% gelatin, and maintained using endothelial cell medium (Sciencell, Carlsbad, CA) containing 1% penicillin/streptomycin, 5% fetal bovine serum (FBS), and 1% endothelial cell growth supplement (ECGS). Cells at passages 3–8 were used for different tests. HUVECs were starved in medium 199 (M199; Welgene, Gyeongsan, Korea) containing 2% FBS before being treated with peptide or antibody.

### HUVEC proliferation assay

Proliferation of HUVECs were determined by counting of Ki67-positive cells^38^. HUVECs (4 × 10^4^ cells/well) were grown on gelatin-coated cover glasses, and treated with N-NAM or scN-NAM in M199 (Welgene, Gyeongsan, Korea) containing 2% FBS for 24 h, and then fixed with 4% paraformaldehyde (PFA; Sigma Aldrich, St. Louis, MO). The fixed cells were blocked with PBS containing 0.1% Trion X-100 and 1% normal goat serum for 30 min and they were incubated with anti-Ki67 antibody (Abcam, Cambridge, UK) overnight at 4 °C. Ki67-positive cell were visualized with using rhodamine conjugated goat anti-rabbit IgG (Jackson ImmunoRes, West Grove, PA). The proliferation rates of HUVECs were calculated by counting Ki67-positive cells (1 mm^2^) in 10 photographs taken from confocal microscopy (Carl Zeiss, Oberkochen, Germany).

### Wound healing assay

HUVECs were seeded in 24-well plates and grown to a 90% confluency. The wounds were made across the center of wells using a yellow tip. After washing with M199, cells were treated with or without peptides in the same medium (M199 containing 2% FBS). Cell migration was assayed using live cell imaging microscope (JuLi Stage; NanoEnTek, Seoul, South Korea) for 12 h. Cell migration into wounds were analyzed using the ImageJ software (https://imagej.nih.gov/ij/download.html) MRI Wound Healing Tool (National Institute of Health (NIH), Bethesda, MD), and the rate of cell migrations were calculated using the following equation: ((area at 0 h − area at 12 h)/area at 0 h) × 100.

### Tube formation assay

Matrigel was added into 48 well (100 µl/well) and polymerized at 37 °C for 30 min. HUVECs (5 × 10^4^) were seeded on Matrigel with or without peptides and with or without co-treating antibodies or sTie2 for 12 h. Tube formation was photographed and calculated by measuring tube numbers and lengths in four different fields per well using a real-time cell history recorder (JuLi stage; NanoEnTek, Seoul, South Korea). Data analysis was performed using the Angiogenesis Tools in ImageJ (https://imagej.nih.gov/ij/download.html) (NIH, Bethesda, MD).

### MTT cell viability assay

HUVEC viability was analyzed using an MTT (3-(4,5-dimethylthiazol-2-yl)-2,5-diphenyl tetrazolium bromide) assay. Briefly, HUVEC cultures were treated with N-NAM or scN-NAM peptide for 24 h; then, 10 μl of an MTT stock solution (5 mg/ml in 0.01 M PBS) was added and incubated for 1 h at 37 °C. Media was removed, and DMSO (200 μl/24-well) was added to solubilize the formazan product. After 30 min at room temperature, the absorbance was measured at 460 nm.

### Matrigel plug assay

Matrigel plug assay was performed as previously described^39^. In brief, Matrigel (0.5 ml/plug) was pre-mixed with 15 units of heparin (Sigma Aldrich, St. Louis, MO). N-NAM (1 µM), scN-NAM (1 µM), or an equivalent volume of PBS was added to the Matrigel mix and the mixture was injected subcutaneously near the ventral midline of 8-week-old BALB/ mice (n = 4 each group). After 12 days, the mice were sacrificed and the plugs were removed. For the measure of hemoglobin (Hb) content, harvested plugs were weighted and incubated overnight in de-ionized water at 37 °C. Then, plugs were crushed with a syringe (26G, Koreavaccine, Gyeonggi-do, South Korea) and centrifuged. Hb content in supernatant was measured using the QuantiChrom Hemoglobin Assay Kit (BioAssay Systems, Hayward, CA) and normalized to plug weight. Neovascularization was determined with 5 µm slice of paraffin-embedded plugs by CD31 immunostaining. Capillary structures were counted by region of interest (ROI) (five ROI per plug; at least five plugs per condition from two separate experiments).

### siRNA transfection

siNinj1 and siCon (control siRNA) (100 pM of each, IDT, Coralville, IA) were individually mixed with Oligofectamine (Invitrogen, Carlsbad, CA) according to the manufacturer’s instructions, and the siRNA-lipid complexes thus produced were added to HUVECs (3 × 10^5^/well) and incubated for 30 h.

### Immunoblot analysis

Brain tissue or HUVECs were homogenized or lysed with RIPA buffer. After centrifugation (12,000×*g* for 15 min), supernatants were collected form samples and protein concentrations of supernatants were determined using a bicinchoninic acid (BCA; ThermoFisher Scientific, Waltham, MA) assay. Protein samples were then applied into 6–12% SDS-PAGE gels and immunoblotted using one or more of the following primary antibodies: anti-angiotensin 1 (Novus, Centennial, CO), anti-angiotensin 2 (Novus, Centennial, CO), anti-endothelial NOS (eNOS) (1:3000; Santa Cruz Biotechnology, Dallas, TX), anti-phospho-eNOS (1:3000; Cell Signaling Technology, Danvers, MA), anti-Ninjurin 1_whole_ (1:2000; BD Biosciences, San Jose, CA), anti-ERK, anti-phospho-ERK, anti-Akt, anti-phospho-Akt (1:2000; Cell Signaling), and anti-GAPDH (1:10,000; Cell Signaling Technology, MA). The membranes were incubated with anti-rabbit HP-conjugated or anti-mouse HP antibody (1:3,000, Millipore, Billerica, MA) and developed using a chemiluminescence kit (ThermoFisher Scientific, Waltham, MA).

### VEGF and MMP-9 ELISA

Cells were grown to subconfluence in gelatin-coated 12-well plates, starved for 2 h, and treated with peptides (50 nM) or antibodies (0.5 µg/ml) for 30 h. Conditioned media were harvested and centrifuged at 8000 rpm. for 2 min at 4 °C to eliminate dead cells, and the supernatants obtained were transferred to clean tubes. VEGF and MMMP-9 levels were measured by ELISA kit (ThermoFisher Scientific, Waltham, MA) according to the manufacturer’s instructions.

### Pull-down assay

Pull-down assays were performed using streptavidin agarose beads (Pierce, Rockford, IL). Briefly, HUVEC lysates were preincubated with anti-Ninj1 antibody (Abcam, Cambridge, UK) with rotation for 15 min at 4 °C and then incubated with biotinylated-N-NAM (25–500 nM) or biotinylated-scN-NAM (bt-scN-NAM, 200–500 nM) for 3 h. These mixtures were then incubated with 30 μl of streptavidin beads for 24 h at 4 °C, centrifuged at 8000 rpm. for 2 min, washed three times, and immunoblotted using anti-Ninj1 (1:1000; Abcam, Cambridge, UK), anti-Na^+^/K^+^ ATPase (Abcam, Cambridge, UK), and Na^+^/H^+^ exchanger (Millipore, Billerica, MA).

### Immunofluorescence staining and IgG staining

For immunofluorescence staining and IgG staining, the rat brain were transcardially perfused with saline, and then the brains tissue were fixed in 4% PFA for 2 days at 4 °C. Brain tissue were coronally sectioned at 30 μm thickness using a vibratome and then immunostained. Briefly, brain sections were washed by PBS and blocked with PBS containing 5% FBS, 5% horse serum, 2% BSA and 0.1% Triton X-100 for 1 h at room temperature. Anti-rat endothelial cell antigen-1 (RECA-1, 1:200) (AbD Serotec, Kidlington, UK) was incubated at 4 °C overnight. Rhodamine labeled anti-rabbit IgG (1:300; Jackson ImmunoRes, West Grove, PA) was used as secondary antibody. The brain sections were then mounted using mounting medium containing DAPI (Vector Laboratories, Peterborough, UK), and endothelial areas were analyzed using AngioTool Software 0.6a (https://angiotool.software.informer.com/0.6/) (National Cancer Institute, Gaithersburg, MD). Rat IgG staining was carried out as previously described^40^. To remove endogenous peroxidase activity, fixed brain section was incubated with 3% H_2_O_2_ in PBS for 30 min and then blocked for 1 h at room temperature. Anti-rat IgG antibody (Vector Laboratories, Peterborough, UK) was used at a concentration of 1: 200 for overnight. After incubation, the brain sections incubated with biotinylated rat IgG antibody for 1 h and then incubated in Vectastain ABC reagent (Vector Laboratories, Peterborough, UK) for 1 h. Using 3,3′-diaminobenzidine (DAB) staining, the reaction product was visualized and the brain section was quantified using Scion image measurement program (https://scion-image.software.informer.com).

### FITC dextran staining

We used FITC-labled dextran (70 kDa) as previously described^41^. At 7 days after MCAO, 70 kDa FITC-dextran (10 mg, Sigma Aldrich, St. Louis, MO) was injected through the tail vein. After 5 min, rat brain was rapidly removed and incubated in 4% paraformaldehyde (PFA; Sigma Aldrich, St. Louis, MO) at 4 °C for 2 days. The rat brains were dissected using vibratome and brain sections were mounted on slide. FITC-dextran images acquired with confocal microscopy (Carl Zeiss, Oberkochen, Germany). Quantitative analysis of the immunofluorescence intensity was carried out using ImageJ (https://imagej.nih.gov/ij/download.html).

### Statistical analysis

Statistical analysis was performed using one-way of variance (ANOVA) followed by Newman Keuls testing. The analyses were performed using PRISM software 5.0 (Graph Pad Software), and results are presented as the means ± SEMs. Statistical difference was accepted for p values.

## Supplementary information

Supplementary Information.
