# Supplementary material for: Ninjurin 1 dodecamer peptide containing the N-terminal adhesion motif (N-NAM) exerts proangiogenic effects in HUVECs and in the postischemic brain
Source: Sci Rep. 2020 Oct 7;10:16656. doi: 10.1038/s41598-020-73340-5 (PMC7542178; doi:10.1038/s41598-020-73340-5)

**SUPPLEMENTARY MATERIAL**

Ninjurin 1 dodecamer peptide containing the N-terminal adhesion motif (N-NAM) exerts proangiogenic effects in HUVECs and in the postischemic brain

# Seung-Woo Kim^1,2,3#^, Hye-Kyung Lee^1,2#^, Song-I Seol^1,2^, Dashdulam Davaanyam^1,2^, Hahnbie Lee^1,2^, Ja-Kyeong Lee^1,2*^

^1^Department of Anatomy, ^2^Medical Research Center, ^3^Department of Biomedical Sciences, Inha University School of Medicine, Inchon, Korea

#

**Key words**: Ninjurin 1, adhesion motif, angiogenesis, MCAO, HUVECs

**Running title**: Proangiogenic effect of N-NAM in HUVECs and the postischemic brain

# ^#^ They are equally contributed

# ^*^Corresponding author:

Ja-Kyeong Lee, Ph.D.

Department of Anatomy, Medical Research Center, Inha University School of Medicine.

Inharo 100, Inchon, 22202, Republic of Korea, Tel, +82-32-860-9893; [jklee@inha.ac.kr](mailto:jklee@inha.ac.kr)

**Supplementary Figure 1**

**
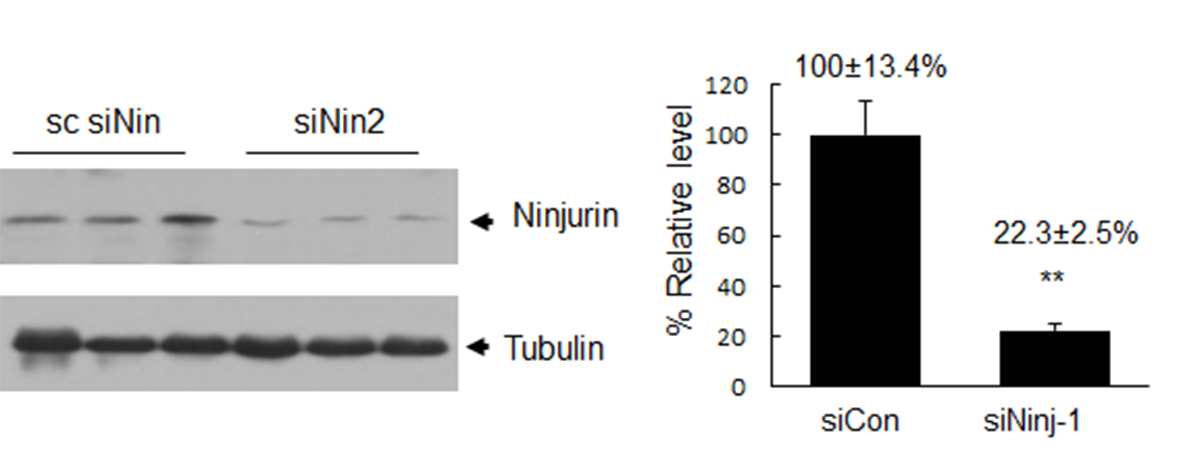
**

**Figure S1. Ninj1 knockdown by Ninj1 siRNA in HUVECs**

Ninj1 siRNA (siNinj1; 100 pM) or control siRNA (si Con; 100 pM) was transfected into HUVECs using Lipofectamine 2000 reagent, according to the manufacturer’s instructions. Protein expression of Ninj1 in the siNinj1 group was significantly reduced compared to the basal level or siCon-group. Results are presented as the means±SEMs (n=3). ** p < 0.01, versus siCon-transfected controls.

**Supplementary Figure 2**

**
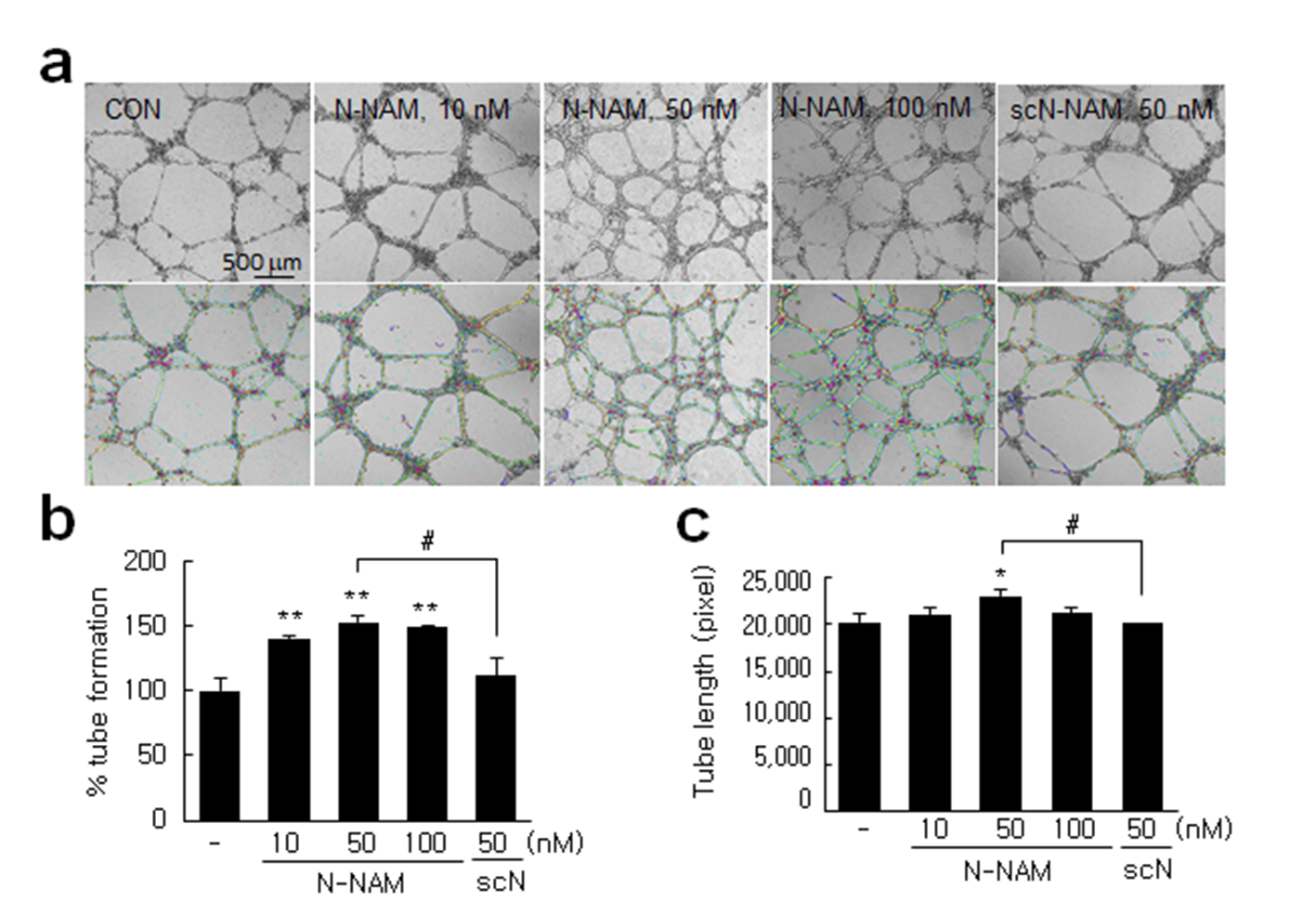
**

**Figure S2. Induction of tube formation by N-NAM in HUVECs**

HUVECs were incubated with N-NAM (10, 50, or 100 nM) or scN-NAM (50 nM) for 12 h, and tube formation was accessed. (**a**) Images obtained using an ImageJ analyzer are presented (green, branches; yellow, master segments; blue, tubes; red, master junctions) and (**b**) the number of tubes and (**c**) total tube lengths were measured. Results are presented as the means±SEMs (n=10). Scale bars, 500 μm. ** *p* < 0.01, **p* < 0.05 versus PBS-treated controls, #*p* < 0.05 between indicated groups.

**Supplementary Figure 3**

**
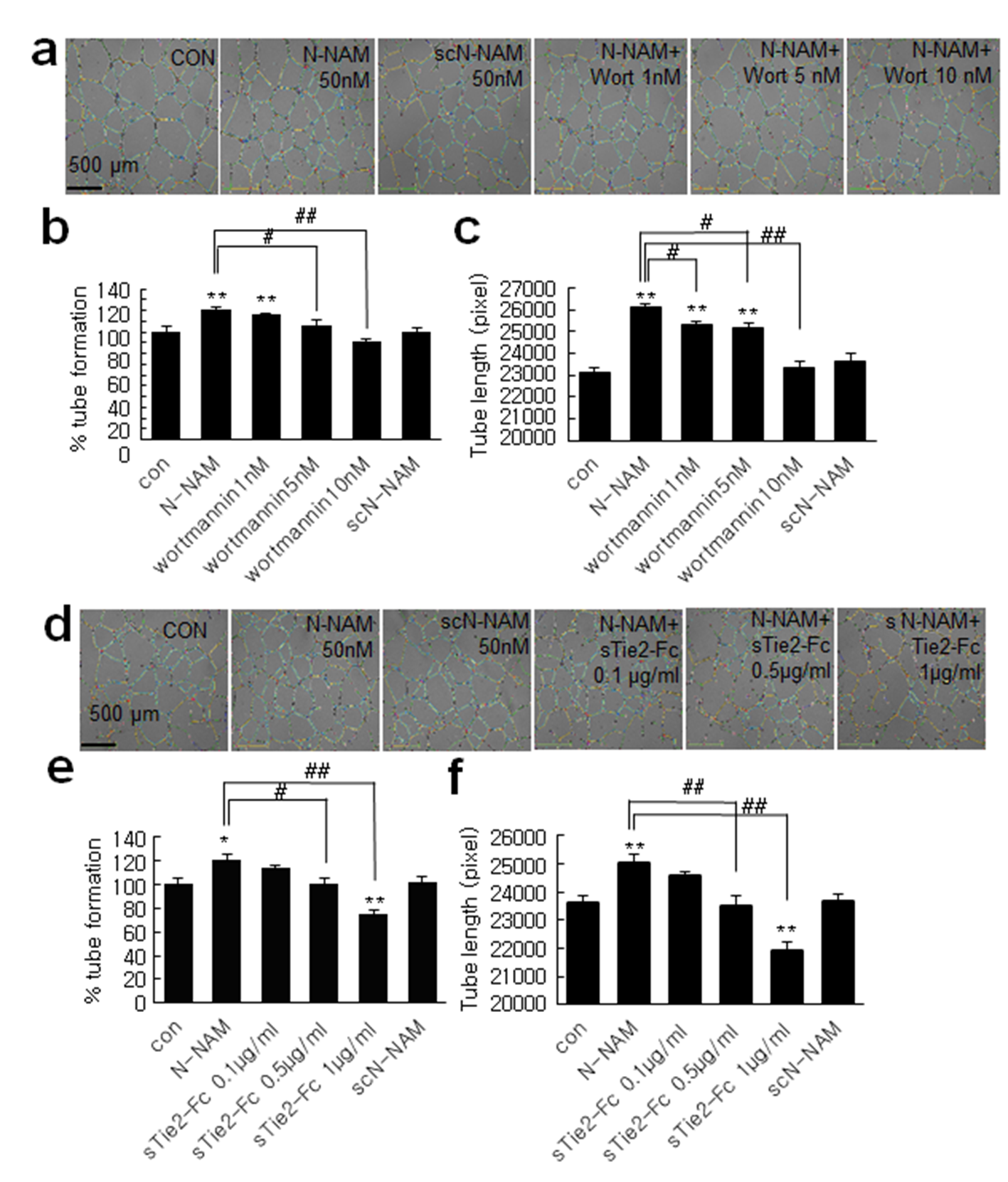
**

**Figure S3. Suppression of N-NAM-mediated tube formation by inhibitor sTie2-Fc and wortmannin in HUVECs**

HUVECs were treated with N-NAM (50 nM) or scN-NAM (50 nM) for 12 h with or without pretreatment with sTie2-Fc (0.1, 0.5, or 1 ug/ml) (**a-c**) or wortmannin (1, 5, or 10 nM) (**d-f**) and tube formation was accessed. Images obtained using an ImageJ analyzer are presented (green, branches; yellow, master segments; blue, tubes; red, master junctions) (**a, c**) and the number of tubes (**b, e**) and total tube lengths (**c, f**) were measured. Results are presented as the means±SEMs (n=10). Scale bars, 500 μm. ** *p* < 0.01, **p* < 0.05 versus PBS-treated controls, ##*p* < 0.01, #*p* < 0.05 between indicated groups.

**Unedited full gel images**


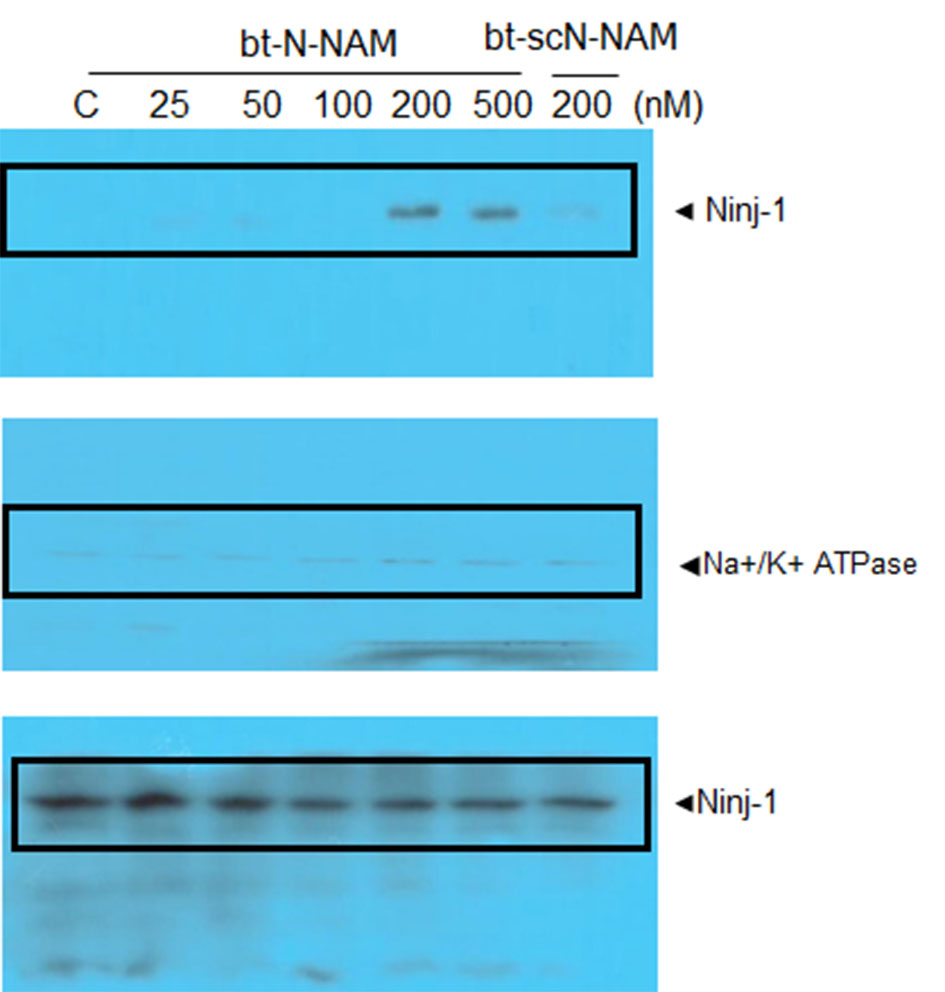


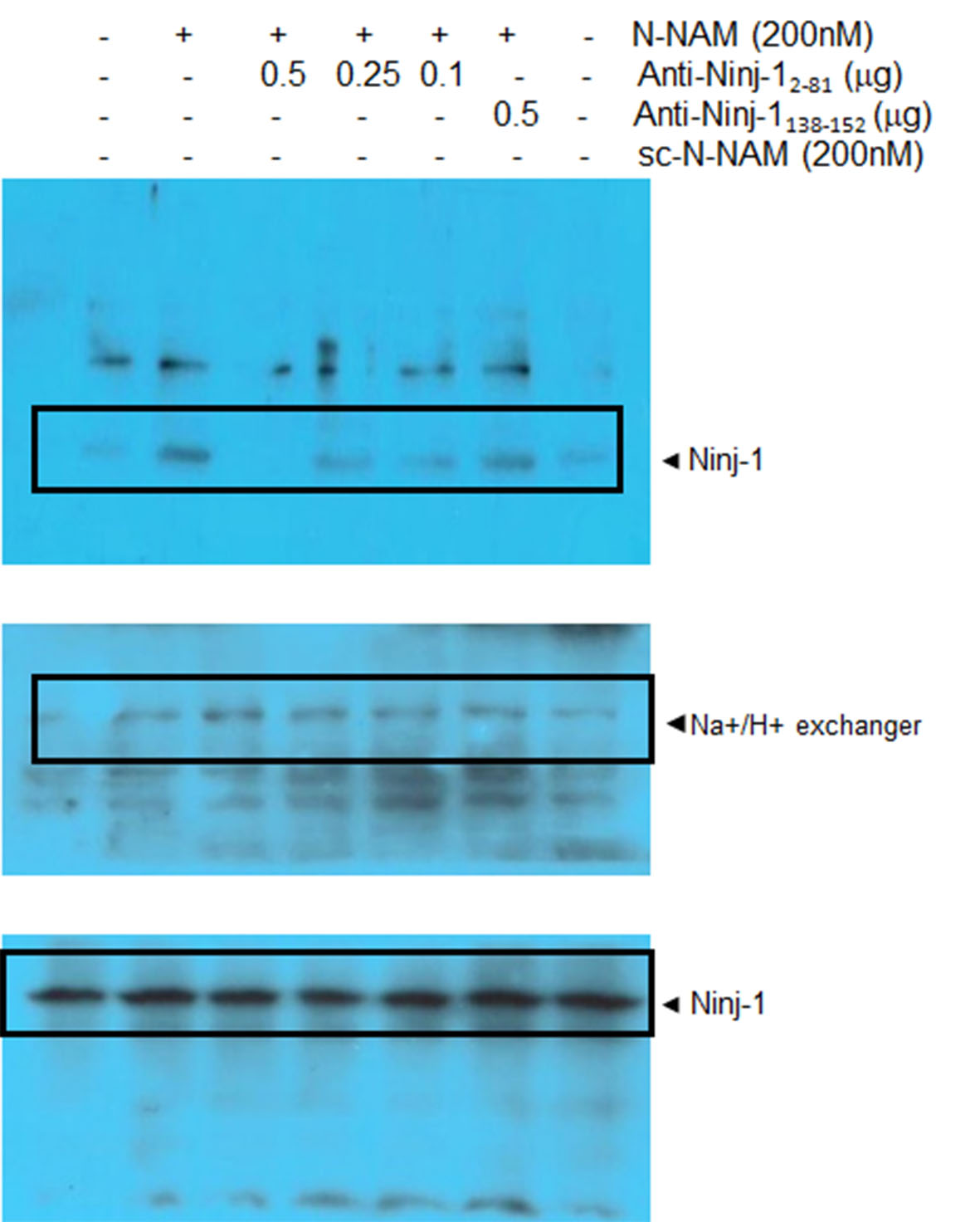


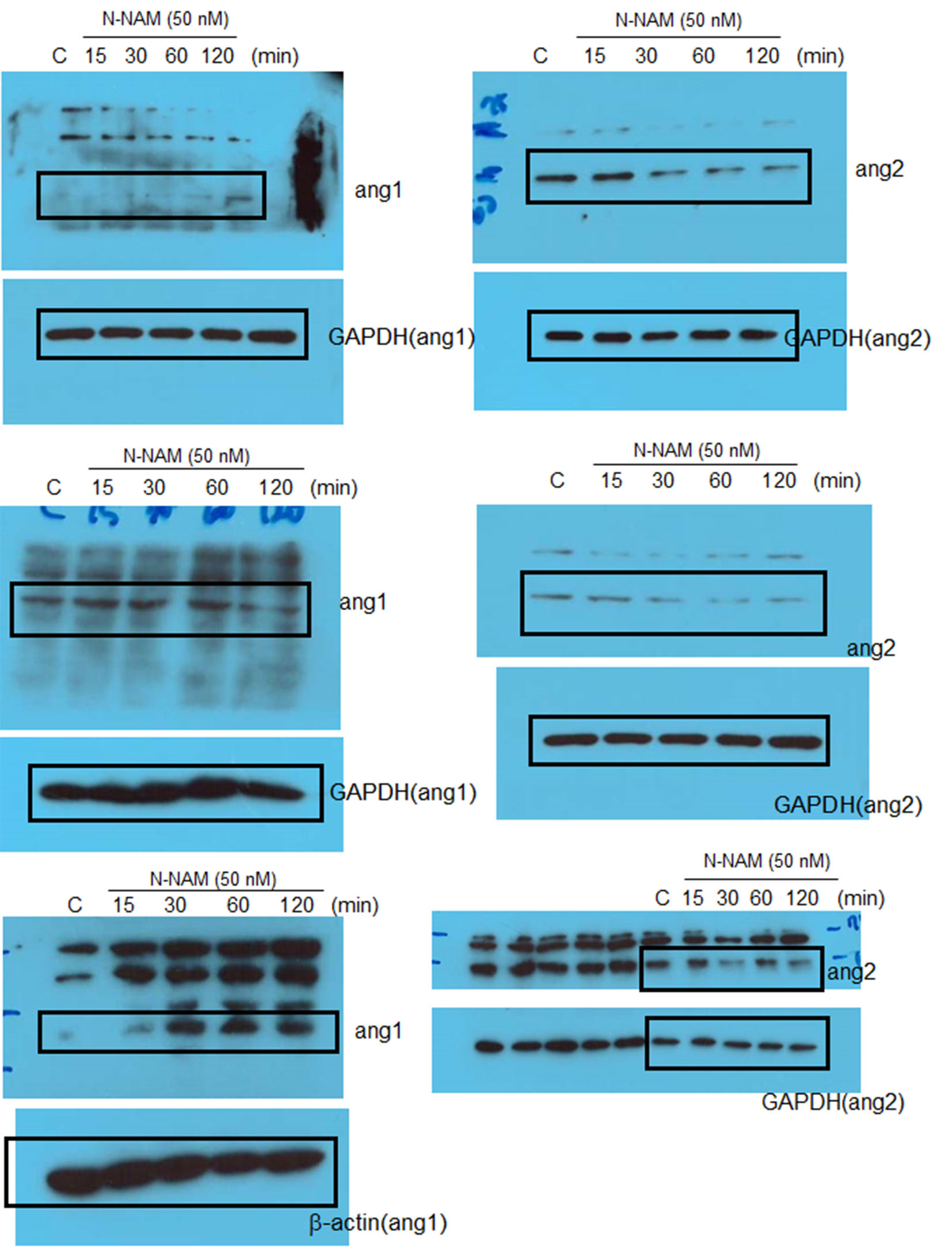


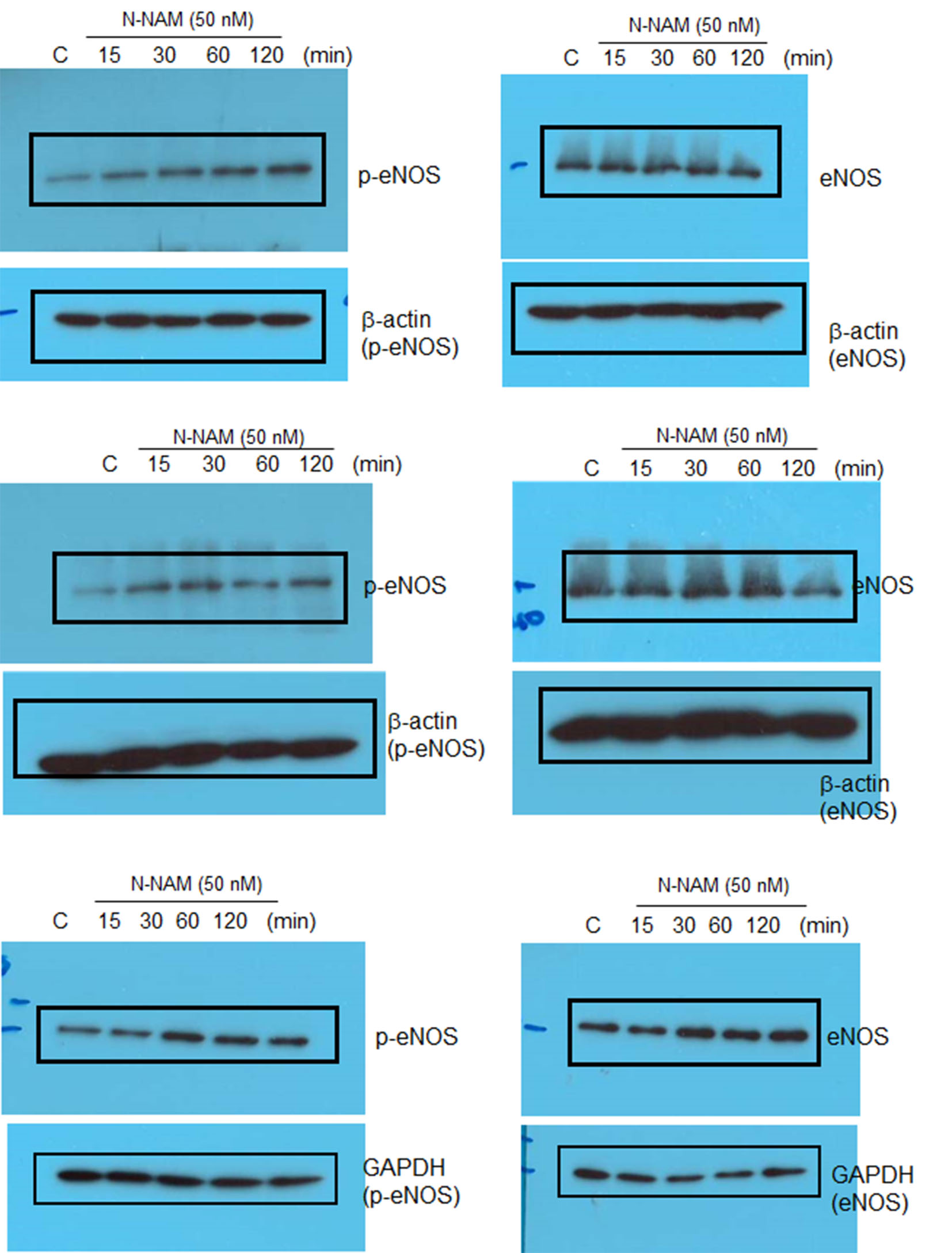


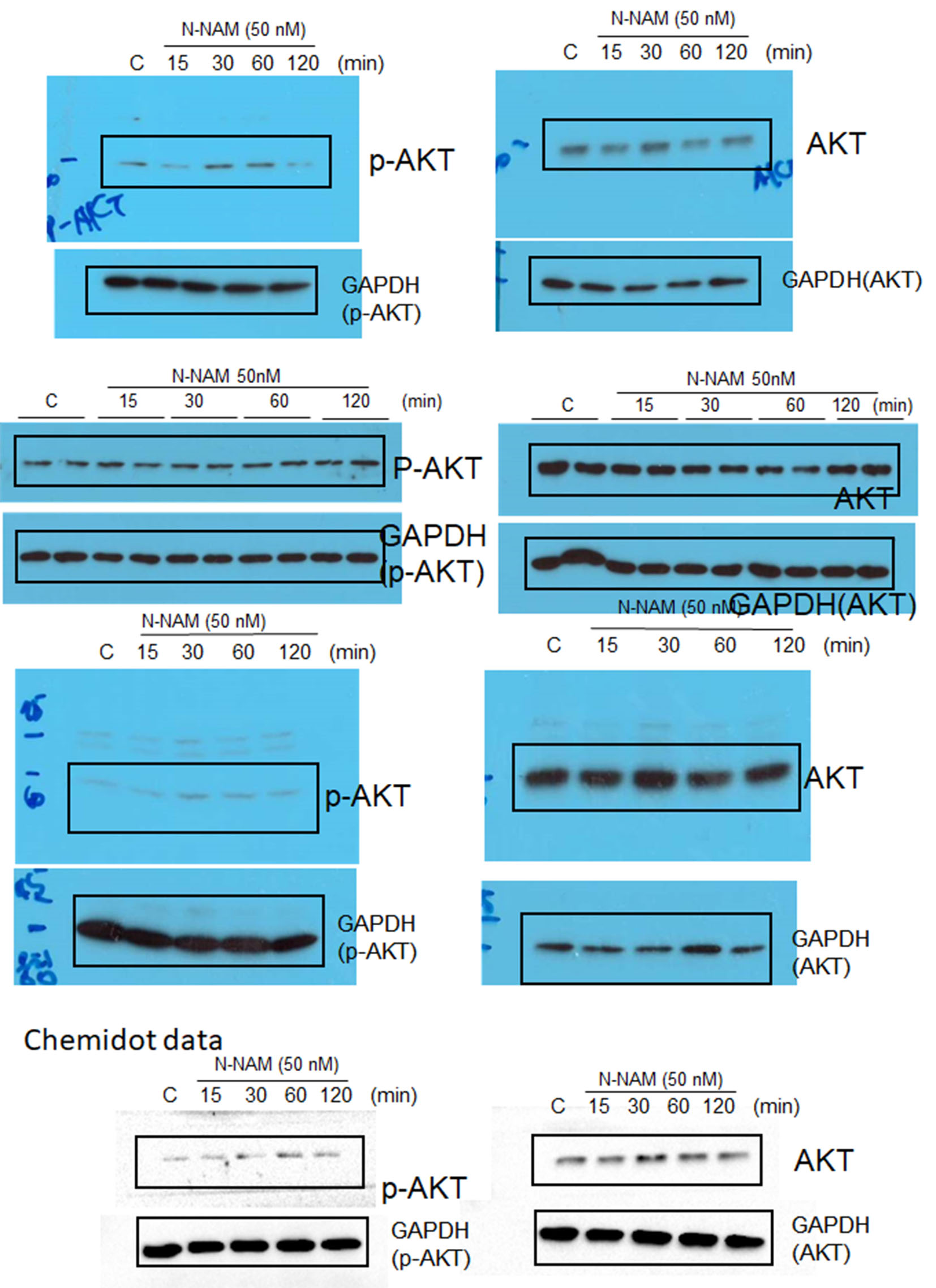


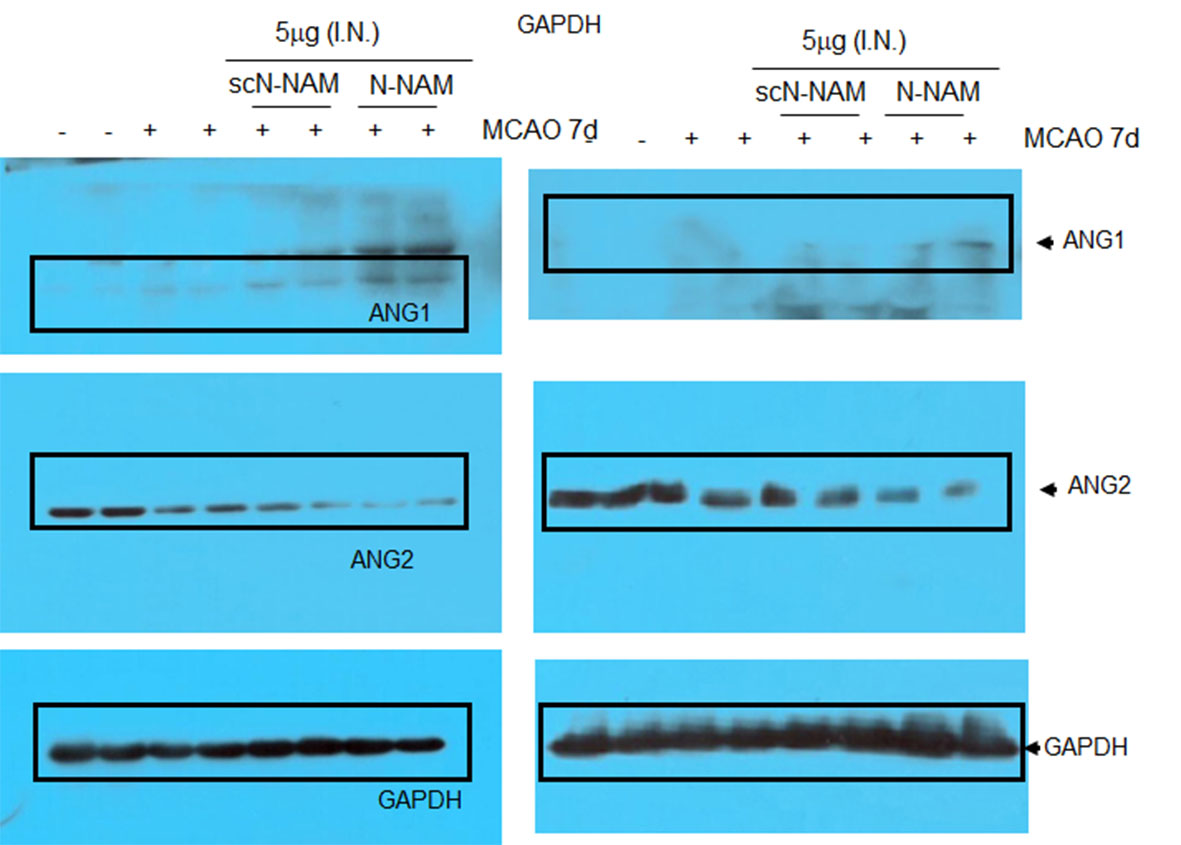

Supplement: Supplementary file 1 — Supplementary Information. [file 41598_2020_73340_MOESM1_ESM.doc]
